# De Novo Biosynthesis of Antidepressant Psilocybin in *Escherichia coli*

**DOI:** 10.1111/1751-7915.70135

**Published:** 2025-04-03

**Authors:** Zhangrao Huang, Yongpeng Yao, Rouyu Di, JianChao Zhang, Yuanyuan Pan, Gang Liu

**Affiliations:** ^1^ State Key Laboratory of Mycology, Institute of Microbiology Chinese Academy of Sciences Beijing People's Republic of China; ^2^ University of Chinese Academy of Sciences Beijing People's Republic of China

**Keywords:** cytochrome P450 enzyme, electron transfer chain, heterologous production, pathway engineering, psilocybin, tryptamine‐derived alkaloid

## Abstract

Psilocybin, a tryptamine‐derived alkaloid, has been granted Breakthrough Therapy designation by the U.S. FDA for treatment‐resistant depression, underscoring its clinical importance. Therefore, sustainable and economic production is urgently needed. Manufacturing of psilocybin in *Escherichia coli* has drawn great attention. However, due to the low expression and activity of the eukaryotic cytochrome P450 enzyme PsiH in the psilocybin biosynthetic pathway, de novo synthesis of psilocybin in prokaryotic cells has been hampered. To overcome this dilemma, we herein demonstrated de novo synthesis of psilocybin in *E. coli* by constructing PsiH variants with N‐terminal domain modifications and expressing the entire biosynthetic pathway at a concordantly low temperature. Improving the supply of precursor and engineering the P450 electron transfer chain resulted in a 33‐fold increase in the titre of norbaeocystin (105.3 mg/L), a key intermediate of psilocybin biosynthesis, and a 17‐fold increase in the titre of psilocybin (14 mg/L). Further enhancement of psilocybin production was achieved by converting norbaeocystin to psilocybin by overexpressing an extra copy of the methyltransferase gene *psiM*. Finally, 79.4 mg/L of psilocybin was produced by optimising flask fermentation conditions, a 100‐fold improvement over the starting strain. Our work demonstrates the successful fungal P450 engineering to improve the catalytic activity in *E. coli* and will advance the sustainable production of the important antidepressant psilocybin in prokaryotic microbial cells.

## Graphical abstract

Our work achieved de novo synthesis of antidepressant psilocybin in a single engineered *Escherichia coli* cell. By constructing the P450 PsiH variants with N‐terminal hydrophobic domain modifications, improving the supply of precursor tryptophan and cofactor NADPH, enhancing the electron transfer (ET) pathway and the final methylation step, and optimising the fermentation conditions, the engineered *E. coli* strain produced psilocybin with a titre of 79.4 mg/L in flask fermentation. Our work provides a sustainable route for fulfilling the industrial production and pharmaceutical application of psilocybin.

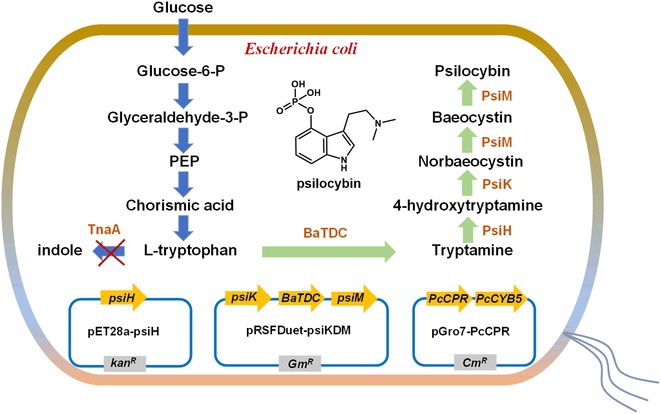

## 1 Introduction

Psilocybin is a tryptamine‐derived alkaloid isolated primarily from the basidiomycete genus *Psilocybe*, the so‐called “magic mushrooms”. The prodrug psilocybin is not psychoactive, but its dephosphorylated derivative psilocin is structurally similar to neurotransmitters, such as serotonin, and can act agonistically on the 5‐HT_2A_ receptor in the human central nervous system (Nichols 2004). Not surprisingly, psilocybin has been used in pharmacological and therapeutic research for the treatment of neuro‐related disorders (Bogenschutz et al. 2015; Carhart‐Harris et al. 2018; Grob et al. 2011). Recent studies have shown that psilocybin has good efficacy in the treatment of depression (Carhart‐Harris et al. 2018; Goodwin et al. 2022; Haikazian et al. 2023), and the Food and Drug Administration (FDA) has granted psilocybin “Breakthrough Therapy” status for the treatment of treatment‐resistant depression, facilitating the drug's marketing. With the pending approval of psilocybin as an antidepressant, efficient and sustainable production methods are urgently needed.

**SCHEME 1:**
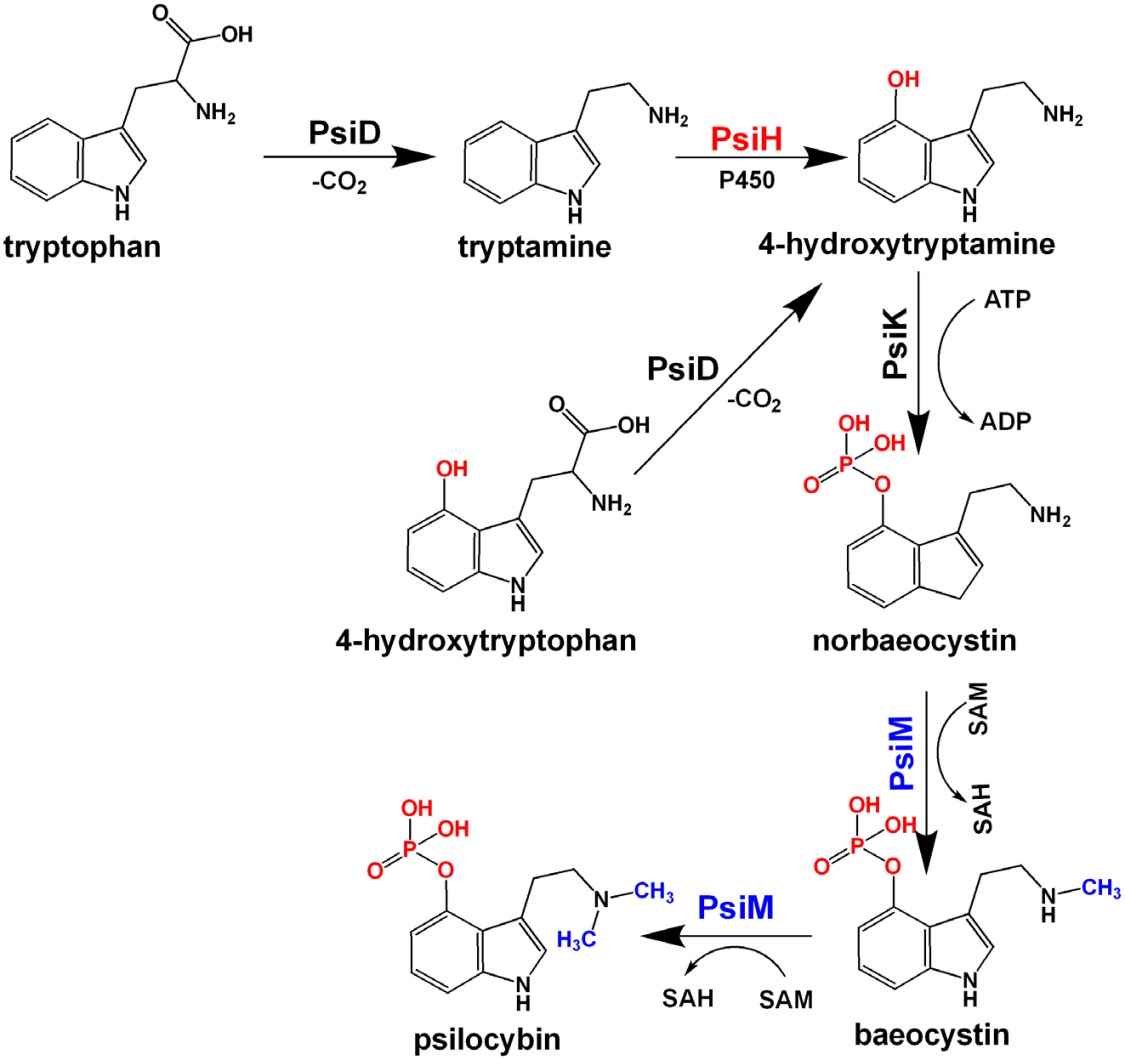
The biosynthetic pathway of psilocybin. PsiD, tryptophan decarboxylase; PsiH, cytochrome P450 monooxygenase; PsiK, phosphate kinase; PsiM, N‐methyltransferase; SAH, *S*‐Adenosylhomocysteine; SAM, *S*‐adenosyl‐L‐methionine.

Biological synthesis of psilocybin is an attractive alternative due to its low‐cost process and high yield. The biosynthetic pathway of psilocybin was first reported in 2017 (Scheme 1) (Fricke et al. 2017). Decarboxylation of tryptophan catalysed by the L‐tryptophan decarboxylase PsiD leads to form tryptamine. Tryptamine is then converted to 4‐hydroxytryptamine by the cytochrome P450 enzyme PsiH, which is a critical rate‐limiting step of psilocybin biosynthesis. The production of 4‐hydroxytryptamine can also be achieved by direct decarboxylation of 4‐hydroxytryptophan catalysed by PsiD (Fricke et al. 2017). Subsequently, a kinase PsiK catalyses the phosphorylation of 4‐hydroxytryptamine to norbaeocystin. Finally, an N‐methyltransferase PsiM catalyses the methylation of norbaeocystin to baeocystin, followed by further methylation to form psilocybin. Elucidation of the psilocybin biosynthetic pathway facilitates its production by in vitro enzymatic synthesis (Fricke et al. 2019). As a membrane‐bound P450 enzyme, PsiH is difficult to be purified. In addition, the PsiH‐catalysed hydroxylation reaction requires a cytochrome P450 reductase (CPR) to transfer two electrons from NADPH, making PsiH‐catalysed hydroxylation impractical in the in vitro process. Using 4‐hydroxytryptophan as the starting substrate, a one‐pot reaction with the PsiD/PsiK/PsiM enzymes was performed without further optimisation, and psilocybin was successfully synthesised in vitro (Fricke et al. 2017). Later, the in vitro procedure was extended to the substrate 4‐hydroxyindole by introducing the tryptophan synthase TrpB, and in a one‐pot reaction with four enzymes, about 20% of the added 4‐hydroxyindole was converted to psilocybin (Blei et al. 2018). However, the production and purification of the enzymes are labour‐intensive and time‐consuming, and the supply of substrates and cofactors is expensive, making in vitro production of psilocybin uneconomical.

Heterologous production of natural bioactive compounds in microbial hosts has achieved great success in recent years (Park et al. 2020; Xu et al. 2020), due to the simple starting substrates, feasible scale‐up and high yields. With the elucidation of the psilocybin biosynthetic pathway, its heterologous production was first implemented in the filamentous fungus *Aspergillus nidulans*. Using the picornavirus' 2A peptide, the biosynthetic genes of psilocybin were polycistronically expressed under the control of a single promoter in *A. nidulans* , and 110 mg/L of psilocybin was obtained by shake flask fermentation (Hoefgen et al. 2018). Recently, through tryptophan catabolism repression, the engineered *A. nidulans* strain produced psilocybin at the titre of 267 mg/L in batch cultures (Janevska et al. 2024). *Saccharomyces cerevisiae* was also successfully engineered to synthesise psilocybin, and production was improved by supplementing the pathway with a CPR from *P. cubensis* (Milne et al. 2020). After optimising fed‐batch fermentation, the titre of psilocybin reached 627 ± 140 mg/L (Milne et al. 2020). As the widely used chassis, *Escherichia coli* is often used for heterologous production of natural products due to its ease of genetic manipulation and ability to grow rapidly to high cell densities on inexpensive media. However, de novo biosynthesis of psilocybin in *E. coli* is challenging due to the low expression and activity of membrane‐bound enzymes (Wagner et al. 2008), such as the P450 enzyme PsiH. To bypass the oxidation step catalysed by PsiH, the tryptophan synthase gene *trpB* was expressed along with the *psiD*, *psiK* and *psiM* genes (Adams et al. 2019). Using 4‐hydroxyindole as a substrate, the titre of psilocybin reached 1.16 g/L via feed‐batch fermentation (Adams et al. 2019). However, the use of 4‐hydroxyindole will increase the cost of commercial production. To circumvent this limitation, an *E. coli* strain expressing the P450 enzyme gene *psiH* was introduced into the fermentation system, and a two‐strain co‐culture system was constructed for the de novo synthesis of psilocybin (Flower et al. 2023). The co‐culture system was able to produce 28.5 ± 0.3 mg/L of psilocybin without supplementation of 4‐hydroxyindole, serine, or methionine (Flower et al. 2023). However, the co‐culture strategy suffers from disadvantages, including the formation of two biomass pools and cell‐to‐cell transport limitations of key intermediates, which limit the overall production metrics (Jones and Wang 2018).

Here, we achieved de novo synthesis of psilocybin in a single engineered *E. coli* cell. By constructing different PsiH variants and expressing the *CPR* gene from *P. cubensis* , the fungal P450 enzyme PsiH was actively expressed in *E. coli* . Then, the entire biosynthetic pathway of psilocybin was successfully reconstituted in *E. coli* to obtain its de novo synthesis in a single cell. The pathway was fully optimised by coordinating the enzyme expression temperature, improving the supply of precursors and cofactors, and engineering the P450 electron transfer (ET) pathway. Finally, the engineered *E. coli* strain produced psilocybin with a titre of 79.4 mg/L by optimising shake‐flask fermentation. Our study provides a sustainable route to produce the important antidepressant psilocybin in microbial cells.

## 2 Experimental Procedures

### 2.1 General Materials

Genes and primers were synthesised by Tsingke Biotechnologies (Beijing, China). Phanta Super‐Fidelity DNA Polymerase (Vazyme) was used for PCR amplification. DNA restriction enzymes were purchased from New England Biolabs (USA). SE seamless cloning and assembly kit (Zoman) was used for in vitro DNA assembly and plasmid construction. Acetonitrile was HPLC grade and was purchased from Sigma‐Aldrich (St. Louis, MO, USA). All other chemicals and solvents were of analytical grade. All buffers and solutions were prepared in Milli‐Q water.

### 2.2 Plasmid Construction for Pathway Reconstitution

To obtain the active P450 enzyme PsiH in *E. coli* , the genes encoding PsiH, cytochrome P450 reductase (PcCPR) and cytochrome b5 (PcCYB5) from *P. cubensis* were codon optimised and synthesised. To improve the expression of *psiH*, the small ubiquitin‐like modifier (SUMO) coding sequence was also codon optimised and synthesised. Then, the wild‐type *psiH* and various *psiH* variants, including the genes encoding the truncated PsiH (trPsiH), SUMO‐PsiH, SUMO‐trPsiH, 5144‐trPsiH and SUMO‐5144‐trPsiH, were constructed and cloned into the *Nde*I and *Xho*I digested plasmid pET28a, respectively, resulting in plasmids pHZR01–6 (Table S1). The *PcCPR* gene was cloned into plasmid pGro7 and polycistronically expressed with the chaperone protein encoding genes *groES* and *groEL* under the control of the *araBAD* promoter, resulting in plasmid pHZR07. To further enhance the activity of PsiH, the *PcCYB5* gene, which encodes the second ET protein of fungal P450s (Durairaj et al. 2016), was further cloned into plasmid pHZR07, resulting in plasmid pHZR08.

To optimise the first biosynthetic step of psilocybin, three L‐tryptophan decarboxylase genes, including *psiD* from *P. cubensis* , *BaTDC* from *Bacillus atrophaeus* and *CrTDC* from *Catharanthus roseus* , were codon optimised and synthesised. They were cloned into the *Nde*I and *Xho*I digested plasmid pRSFDuet‐GmR, which was derived from pRSFDuet‐1 by changing the kanamycin resistance gene to the gentamicin resistance gene, resulting in plasmids pHZR09–11, respectively.

To reconstitute the biosynthetic pathway of psilocybin in *E. coli* , the *psiK* and *psiM* genes from *P. cubensis* were codon optimised and synthesised. They were ligated to the two cloning sites of plasmid pRSFDuet‐GmR, resulting in plasmid pHZR12. The *psiD* gene was then cloned into pHZR12, resulting in plasmid pHZR13.

To improve the supply of cofactor NADPH for the PsiH‐catalysed hydroxylation reaction, the *nadK* gene from *E. coli* was amplified and cloned into the plasmid pCDFDuet‐Amp, which was derived from pCDFDuet‐1 by changing the streptomycin resistance gene to the ampicillin‐resistant gene, resulting in plasmid pHZR14. To improve the supply of substrate *S*‐adenosyl‐L‐methionine (SAM) for the PsiM‐catalysed methylation reaction, the *metK* gene was amplified from *E. coli* , and the *SAM2* gene from *Saccharomyces cerevisiae* was codon optimised and synthesised. They were then cloned into plasmid pHZR14, resulting in plasmids pHZR15 and pHZR16, respectively. The constructed plasmids were transformed into *E. coli* strain DH5α for propagation and sequenced for further expression in *E. coli* strain BL21 (DE3). All plasmids constructed in this work are listed in Table S1, and the primers used in this study are listed in Table S2; the codon‐optimised sequences expressed in this work are shown in Table S4.

### 2.3 Gene Knockout Using CRISPR/Cas9‐Mediated Gene Editing Strategy

The CRISPR/Cas9‐mediated gene editing strategy was used to knock out the *trpR* and *tnaA* genes in *E. coli* BL21. The plasmid pCas‐prha‐NEW for expressing *cas9* was transformed into *E. coli* BL21. Then, 10 mM of L‐arabinose was added for λ‐Red induction, and *E. coli* BL21 electroporation‐competent cells harbouring pCas‐prha‐NEW were prepared (Sharan et al. 2009). For electro‐transformation, 50 μL of cells were mixed with 100 ng of pTarget series and 400 ng of donor DNA (fragment containing 500 bp up‐ and downstream of the target genes). Electroporation was performed in a 1 mm Gene Pulser cuvette (Bio‐Rad) at 2.5 kV, and the product was immediately suspended in 1 mL of ice‐cold LB medium. Cells were recovered at 30°C for 1 h, and transformants were selected on LB agar containing kanamycin (50 μg/mL) and spectinomycin (50 μg/mL) overnight at 30°C. The knockout strains were further verified by colony PCR and DNA sequencing.

To cure the pTarget series, the knockout strain carrying both pCas‐prha‐NEW and the pTarget series was inoculated in 2 mL of LB medium containing kanamycin (50 μg/mL) and L‐rhamnose monohydrate (10 mM) for 16 h. The cells were then diluted and selected on LB plates containing kanamycin (50 μg/mL). Colonies were confirmed as cured by determining their sensitivity to spectinomycin (50 μg/mL). The cured colonies from the pTarget series were used in a second round of genome editing. Finally, pCas‐prha‐NEW was cured by growing the colonies in LB medium without any antibiotics overnight at 37°C.

### 2.4 Characterisation of Key Enzymes via In Vivo Biotransformation

To verify the expression level of different *psiH* variants, plasmids pGro7 and each of pHZR01–6 were co‐transformed into *E. coli* BL21 (DE3). Transformants were selected on the LB agar plates supplemented with 50 μg/mL kanamycin and 25 μg/mL chloramphenicol at 37°C overnight. The colony was then picked and cultured in 3 mL of LB medium at 37°C overnight. The incubated cells were then transferred to 100 mL of fresh LB medium (1% v/v) supplemented with 50 μg/mL kanamycin and 25 μg/mL chloramphenicol. When the OD_600_ of the culture reached approximately 0.4 ~ 0.6, 0.1 mM IPTG and 500 μg/mL L‐arabinose were added to induce protein expression for 14 h at 20°C. 3 mL of cells were then collected and resuspended in 1 mL Tris–HCl buffer (50 mM, pH 7.5). Total cell lysis was used to detect protein expression by SDS‐PAGE analysis.

To test the catalytic ability of different PsiH variants, plasmids pHZR07 and each of pHZR01–6 were co‐transformed into *E. coli* BL21 (DE3), resulting in strains H1–6. The strains were cultured, and proteins were expressed as described above. 50 mL of cells were harvested by centrifugation. Then, 1 mL of the fresh LB medium supplemented with 500 μg/mL tryptamine was added, and the cells were further cultured for 1 day at 20°C. To verify the catalytic ability of different L‐tryptophan decarboxylases, plasmids pHZR09–11 were transformed into *E. coli* BL21 (DE3), resulting in strains D1–3. After the protein was expressed, 50 mL of cells were harvested by centrifugation. Then, 1 mL of the fresh LB medium supplemented with 500 μg/mL of L‐tryptophan was added, and the cells were further cultured for 1 day at 20°C. To verify the catalytic ability of PsiK and PsiM, the plasmid pHZR12 was transformed into *E. coli* BL21 (DE3), resulting in strain KM. After the protein was expressed, 50 mL of cells were harvested by centrifugation. Then, 800 μL of PsiH‐transformed products and 200 μL of the fresh LB medium were added, and the cells were further cultured for 1 day at 20°C. After the in vivo transformation, 500 μL methanol was added to 500 μL of the cell mixture. The mixture was vortexed and centrifuged at 12,000 rpm for 10 min. The supernatant was then used for HPLC analysis.

### 2.5 Reconstitution of Psilocybin Biosynthetic Pathway in *E. coli*

To achieve de novo biosynthesis of psilocybin in *E. coli* , the plasmids pHZR02, pHZR07 and pHZR13 were co‐transformed into *E. coli* BL21 (DE3), resulting in strain P03. Transformants were selected on the LB agar plate supplemented with 50 μg/mL kanamycin, 25 μg/mL chloramphenicol and 40 μg/mL gentamicin at 37°C overnight. The colony was then picked and cultured in 3 mL of LB medium at 37°C overnight. Subsequently, the incubated cells were transferred to 100 mL of the fresh LB medium (1% v/v) supplemented with antibiotics as described above. When the OD_600_ of the culture reached approximately 0.4 ~ 0.6, 0.1 mM IPTG and 500 μg/mL L‐arabinose were added. The cells were further cultured at 20°C, 220 rpm for 24 h.

To detect the product, 500 μL methanol was added to 500 μL of the cell mixture. The mixture was vortexed and centrifuged at 12,000 rpm for 10 min. The supernatant was then used for HPLC analysis. To further improve psilocybin production, additional strains were constructed and tested, and they are listed in Table 1. All the proteins expressed in this study are listed in Table S3.

**TABLE 1:** Strains used and generated in the study.

Strain	Genotype	Plasmid	Reference
H1	*psiH, PcCPR*	pHZR01, pHZR07	This study
H2	*trpsiH, PcCPR*	pHZR02, pHZR07	This study
H3	*5144C1NTD‐trpsiH, PcCPR*	pHZR03, pHZR07	This study
H4	*SUMO‐trpsiH, PcCPR*	pHZR04, pHZR07	This study
H5	*SUMO‐psiH, PcCPR*	pHZR05, pHZR07	This study
H6	*SUMO‐5144C1NTD‐psiH, PcCPR*	pHZR06, pHZR07	This study
D1	*psiD*	pHZR09	This study
D2	*BaTDC*	pHZR10	This study
D3	*CrTDC*	pHZR11	This study
KM	*psiK, psiM*	pHZR12	This study
P01	△tnaA		This study
P02	△tnaA △trpR		This study
P03	*trpsiH, psiK, BaTDC, psiM, PcCPR*	pHZR02, pHZR07, pHZR13	This study
P04	*trpsiH, psiK, BaTDC, psiM, PcCPR, PcCYB5*	pHZR02, pHZR08, pHZR13	This study
P05	*trpsiH, psiK, BaTDC, psiM, nadK, PcCPR, PcCYB5*	pHZR02, pHZR08, pHZR13, pHZR14	This study
P06	△tnaA, *trpsiH, psiK, BaTDC, psiM, nadK, PcCPR, PcCYB5*	pHZR02, pHZR08, pHZR13 and pHZR14	This study
P07	△tnaA △trpR, *trpsiH, psiK, BaTDC, psiM, nadK, PcCPR, PcCYB5*	pHZR02, pHZR08, pHZR13 and pHZR14	This study
P08	△tnaA △trpR, *trpsiH, psiK, BaTDC, psiM, nadK, metK, PcCPR, PcCYB5*	pHZR02, pHZR08, pHZR13 and pHZR15	This study
P09	△tnaA △trpR, *trpsiH, psiK, BaTDC, psiM, nadK, SAM2, PcCPR, PcCYB5*	pHZR02, pHZR08, pHZR13 and pHZR16	This study
P10	△tnaA △trpR, *trpsiH, psiK, BaTDC, PacPsiM, nadK, PcCPR, PcCYB5*	pHZR02, pHZR08, pHZR17 and pHZR14	This study
P11	△tnaA △trpR, *trpsiH, psiK, BaTDC, GdPsiM, nadK, PcCPR, PcCYB5*	pHZR02, pHZR08, pHZR18 and pHZR14	This study
P12	△tnaA △trpR, *trpsiH, psiK, BaTDC, PscPsiM, nadK, PcCPR, PcCYB5*	pHZR02, pHZR08, pHZR19 and pHZR14	This study
P13	△tnaA △trpR, *trpsiH, psiK, BaTDC, 2 × psiM, nadK, PcCPR, PcCYB5*	pHZR20, pHZR08, pHZR13 and pHZR14	This study

*Note:* All the strains were engineered from *Escherichia. coli* BL21 (DE3).

### 2.6 Optimisation of Fermentation Medium

To test psilocybin could be de novo synthesised in strain P13, a modified M9 medium was used for fermentation. The modified M9 medium contained 17.4 g/L Na_2_HPO_4_·7 H_2_O, 3 g/L KH_2_PO_4_, 1 g/L NH_4_Cl, 0.5 g/L NaCl, 0.49 g/L MgSO_4_·7H_2_O, 0.11 g/L CaCl_2_ and 20 g/L glucose. The colony of strain P13 on the LB plate was selected and cultured in 3 mL of M9 medium at 37°C overnight. Subsequently, the incubated cells were transferred to 100 mL of M9 medium in a baffled flask (500 mL) supplemented with the corresponding antibiotics. When the OD_600_ of the culture reached approximately 0.4 ~ 0.6, 0.1 mM IPTG and 500 μg/mL L‐arabinose were added. The cells were further cultured at 20°C, 220 rpm for 120 h. At every 12‐h interval, 0.5 mL of the broth was collected and psilocybin production was determined. To further improve psilocybin production, the optimal fermentation mediums, including fresh LB, LBG (LB with 2% glucose) and TB medium, were investigated.

### 2.7 Analytical Methods

High performance liquid chromatography (HPLC) analysis was performed on a Waters 2695 system (USA) using a C18 analytical column (Gemini 250 × 4.6 mm, particle size 5 μm; Phenomenex). The mobile phase flow rate was 1 mL/min, and the column temperature was 25°C. The analytical method is a linear gradient of 5%–19% acetonitrile (MeCN)‐H_2_O (v/v, 0.1% formic acid) for 12 min, followed by 100% MeCN for 4 min and rebalanced by 5% MeCN (v/v, 0.1% formic acid) for 6 min. Using this method, the following retention times were observed: norbaeocystin (5.92 min), baeocystin (6.41 min), psilocybin (6.75 min), 4‐hydroxytryptamine (6.95 min), norpsilocin (7.25 min), psilocin (7.93 min), tryptamine (8.96 min) and tryptophan (10.00 min).

For the liquid chromatograph‐high resolution mass spectrometer (LC‐HRMS) analysis, liquid chromatography (LC) separation was performed on an Agilent 1260 series system (Agilent, USA) with a C18 analytical column (Gemini 250 × 4.6 mm, particle size 5 μm; Phenomenex). The LC analytical method is the same as described above. Mass spectra were performed on an Agilent Accurate‐Mass‐Q‐TOF MS 6520 system equipped with an electrospray ionisation (ESI) source in positive mode. The LC‐HRMS data were recorded and analysed using MassHunter Workstation software (version B.04.00, Agilent, Santa Clara, CA, USA).

The chemical compounds psilocybin (Cayman), tryptamine (Macklin) and tryptophan (Macklin) were purchased as authentic standards, and their standard curves were obtained by HPLC analysis. The peak area of the HPLC trace was recorded under 280 nm for all compounds. The concentration of the authentic standards was set as x, and the peak area of the HPLC trace was set as y, resulting in a regression equation (Figure S10). Due to limited commercial availability and extremely high cost, baeocystin, norbaeocystin and 4‐hydroxytryptamine were quantified using the standard curve of their analogues (Adams et al. 2019). Baeocystin and norbaeocystin were quantified using the standard curve of psilocybin. 4‐hydroxytryptamine was quantified from the standard curve of 5‐hydroxytryptamine.

## 3 Results

### 3.1 Construction of Chimeric PsiH to Improve Its Expression in *E. coli*

**FIGURE 1:**
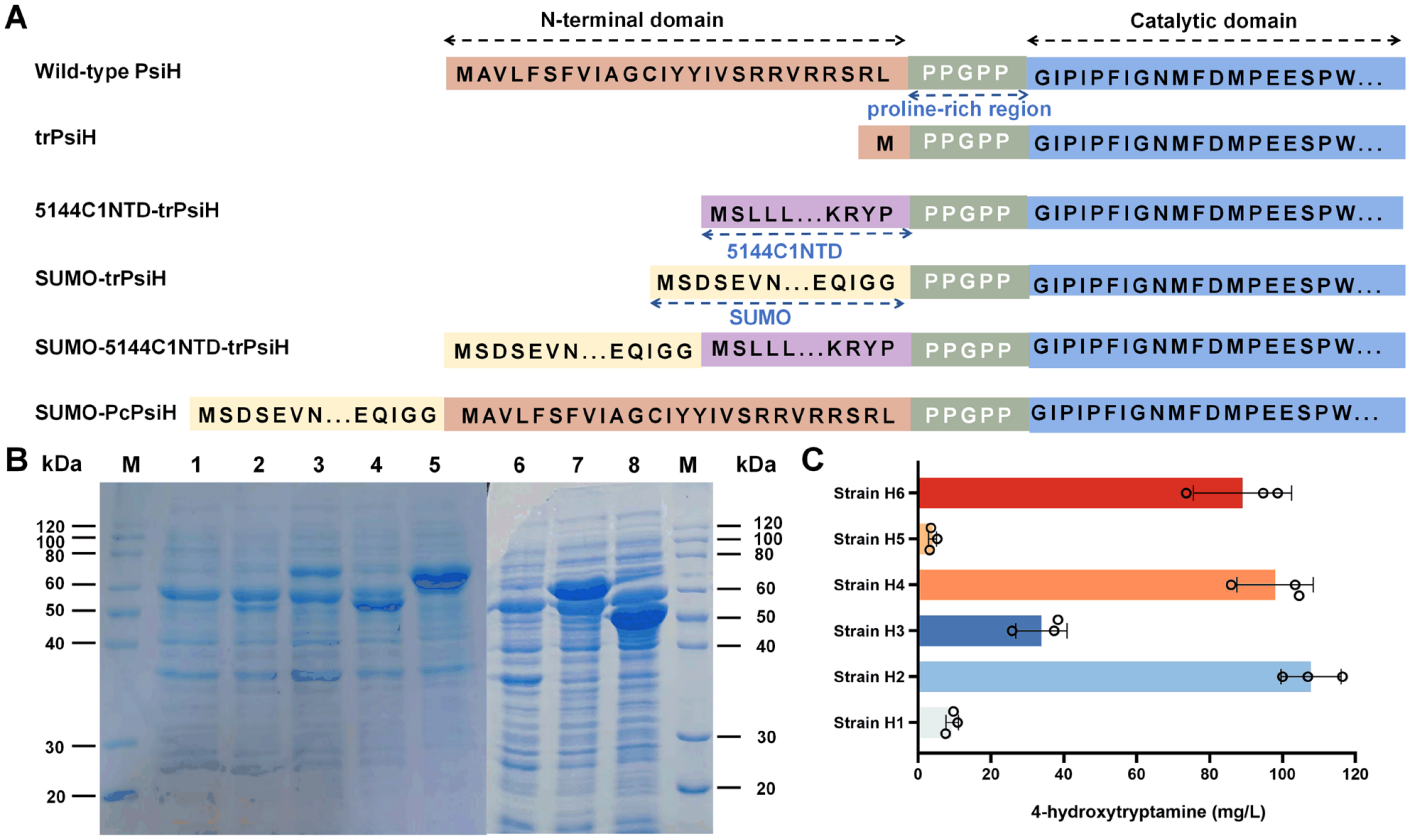
Engineering the P450 monooxygenase PsiH. (A) Sequence structures of various PsiH variants. The N‐terminal hydrophobic region and the C‐terminal catalytic functional domain of PsiH were separated by a proline‐rich region (PPGPP). (B) SDS‐PAGE analysis of the total cell lysis from the *Escherichia coli* BL21 strains expressing different PsiH variants with the molecular chaperones GroES/GroEL. M: Protein marker; lines 1 and 6: Proteins from the *E. coli* BL21 strain carrying the empty plasmid; line 2: PsiH (57.8 kDa) from strain H1; line 3: SUMO‐PsiH (69.1 kDa) from strain H5; line 4: 5144C1NTD‐trPsiH (56.9 kDa) from strain H3; line 5: SUMO‐5144C1NTD‐trPsiH (68.2 kDa) from strain H6; line 7: SUMO‐trPsiH (66.0 kDa) from strain H4; and line 8: TrPsiH (54.7 kDa) from strain H2, respectively. (C) Production of 4‐hydroxytryptamine produced by the *E. coli* strains expressing different *psiH* variants. Strains H1–6 were used for expressing *psiH*, *trPsiH*, *5144C1NTD‐trPsiH*, *SUMO‐trPsiH*, *SUMO‐PsiH* and *SUMO‐5144C1NTD‐trPsiH*, respectively.

The hydroxylation of tryptamine catalysed by the P450 enzyme PsiH is the rate‐limiting step for the biosynthesis of psilocybin in *E. coli* . Due to the membrane‐bound nature of PsiH and the need for enzymatic redox partners for catalysis, de novo synthesis of psilocybin in *E. coli* has been hindered (Adams et al. 2019; Flower et al. 2023). Most eukaryotic P450 enzymes have N‐terminal transmembrane regions that are natively located in the membranes of the endoplasmic reticulum or mitochondrion (Durairaj and Li 2022). Therefore, the expression of eukaryotic P450s in *E. coli* often leads to the aggregation of insoluble proteins (Hausjell et al. 2018). Previous studies have shown that N‐terminal modifications could improve the expression of eukaryotic P450s in *E. coli* , such as deleting the hydrophobic domain (Park et al. 2014) or replacing the hydrophobic domain with the N‐terminal sequence of other P450s (Ichinose et al. 2015; Ichinose and Wariishi 2013). PsiH has an N‐terminal transmembrane region of 19 amino acids (Figure S1), and its C‐terminal catalytic domain is separated from the N‐terminal hydrophobic domain by a proline‐rich region (PPGPP) (Figure 1A). Using this region as a boundary, the N‐terminal hydrophobic region and the C‐terminal catalytic functional domain of PsiH were separated, and several chimeric PsiH proteins were constructed (Figure 1A). We deleted the N‐terminal domain (shown in red) of the wild‐type PsiH to generate the truncated PsiH protein (trPsiH). We also replaced the N‐terminal domain of the wild‐type PsiH with the corresponding domain of CYP5144C1, which has been reported to enhance the expression of several fungal P450s in *E. coli* (Ichinose et al. 2015), to form the chimeric protein 5144C1NTD‐trPsiH. In addition, we also constructed chimeric proteins SUMO‐trPsiH, SUMO‐5144C1NTD‐trPsiH and SUMO‐PsiH (Figure 1A) because the small ubiquitin‐like modifier (SUMO) is a widely used tag sequence to enhance protein expression in *E. coli* (Peroutka Iii et al. 2011). The chimeric proteins were expressed respectively based on the medium copy number plasmid pET28a in *E. coli* BL21 along with the molecular chaperones GroES/GroEL, which could facilitate the proper folding of eukaryotic P450s to obtain the active enzymes (Ahn et al. 2004; Ichinose et al. 2015). The expressed proteins in total cell lysate were then detected by SDS‐PAGE analysis. The results showed that all the PsiH variants were successfully expressed (Figure 1B), but they cannot be purified due to their insolubility. In terms of expression level, all the chimeric proteins have a higher expression level compared to the wild‐type PsiH (Figure 1B, line 2). Amongst them, the expression level of SUMO‐5144C1NTD‐trPsiH, SUMO‐trPsiH and trPsiH was significantly improved (Figure 1B, lines 5, 7 and 8). These results demonstrated that both deletion of the N‐terminal hydrophobic domain and addition of the SUMO sequence promoted the expression of the PsiH protein.

To test the catalytic ability of the chimeric PsiH proteins, the *PcCPR* gene from *P. cubensis* was cloned into the low copy number plasmid pGro7 and was polycistronically expressed with *groEL* and *groES* in *E. coli* . After the substrate tryptamine was fed to the engineered *E. coli* strains (Table 1), the bio‐transformed product 4‐hydroxytryptamine was detected by HRMS analysis (Figure S2B) and further quantified by HPLC analysis (Figure S2A). As shown in Figure 1C, all the strains produced 4‐hydroxytryptamine. Amongst them, the strain H2 expressing *trPsiH* produced the highest titre of 4‐hydroxytryptamine at 107.82 mg/L, which was 10.53‐fold higher than the production of 9.35 mg/L from the strain H1 expressing the wild‐type *psiH*. In addition, the strain H4 expressing SUMO‐trPsiH and strain H6 expressing SUMO‐5144C1NTD‐trPsiH produced 4‐hydroxytryptamine at 97.96 mg/L and 88.97 mg/L, respectively, which were 9.48‐ and 8.52‐fold higher than that of the strain H1. These results indicated that the production of 4‐hydroxytryptamine was positively related to the expression level of the chimeric PsiH proteins. Since the strain H2 expressing *trPsiH* produced the highest titre of 4‐hydroxytryptamine, trPsiH was chosen to reconstitute the psilocybin biosynthetic pathway in *E. coli* .

We also determined the effect of culture temperature on the catalytic ability of trPsiH. Expression of trPsiH was induced at 18°C, 20°C, 22°C, 25°C and 28°C, respectively. By feeding tryptamine, the production of 4‐hydroxytryptamine was quantified by HPLC analysis. The results showed that the strain H2 cultured at 18°C, 20°C and 22°C gave a comparable amount of 4‐hydroxytryptamine (Figure S2C), while the production of 4‐hydroxytryptamine decreased sharply when the strain H2 was cultured at 25°C, indicating that trPsiH might have a good expression level and catalytic efficiency in the range of 18 to 22°C.

### 3.2 Reconstruction of Psilocybin Biosynthetic Pathway in *E. coli*

Having obtained the chimeric PsiH with high catalytic performance, we sought to reconstruct the entire biosynthetic pathway of psilocybin in *E. coli* . Decarboxylation of tryptophan catalysed by PsiD is the first step of psilocybin biosynthesis, and sufficient precursor supply of tryptamine is crucial to improve psilocybin production. Since the L‐tryptophan decarboxylases BaTDC from *B. atrophaeus* (Choi et al. 2021) and CrTDC from *C. roseus* (Noé et al. 1984) showed relatively low *Km* values towards L‐tryptophan and high turnover numbers, their coding genes were optimised and synthesised together with the *psiD* gene from *P. cubensis* . They were cloned into the plasmid pRSFDuet‐GmR and were expressed in *E. coli* BL21. By feeding tryptophan, tryptamine was produced in all three strains (Figure S3A), confirming that all three decarboxylases could convert tryptophan to tryptamine. We further performed the in vivo conversion using the enzymes expressed at different temperatures in the range of 18°C–37°C. We found that the titre of tryptamine from the strain expressing *CrTDC* decreased dramatically with increasing temperature, but the strain expressing *psiD* gave a relatively stable tryptamine production (Figure S3B). Meanwhile, the strain expressing *BaTDC* gave the highest titre of tryptamine at 20°C, which was improved by 45.8% compared with that of the strain expressing *psiD*. Therefore, BaTDC could be a preferred candidate for the reconstruction of the psilocybin biosynthetic pathway in *E. coli* .

Since the expression levels of *psiK* and *psiM* are crucial for the efficiency of psilocybin synthesis, we expressed the *psiM* and *psiK* genes based on the high copy number plasmid pRSFDuet‐GmR in *E. coli* BL21. By adding 4‐hydroxytryptamine‐containing supernatant, which was prepared from the tryptamine‐fed strain H2, the final product psilocybin was detected compared to the negative control and authentic standard (Figure S4A), demonstrating that PsiM and PsiK could convert 4‐hydroxytryptamine to psilocybin. We further expressed *psiM* and *psiK* at different temperatures ranging from 18°C to 37°C. We found that the titre of psilocybin increased with the increase in culture temperature and reached the highest titre of psilocybin at 37°C (Figure S4B).

**FIGURE 2:**
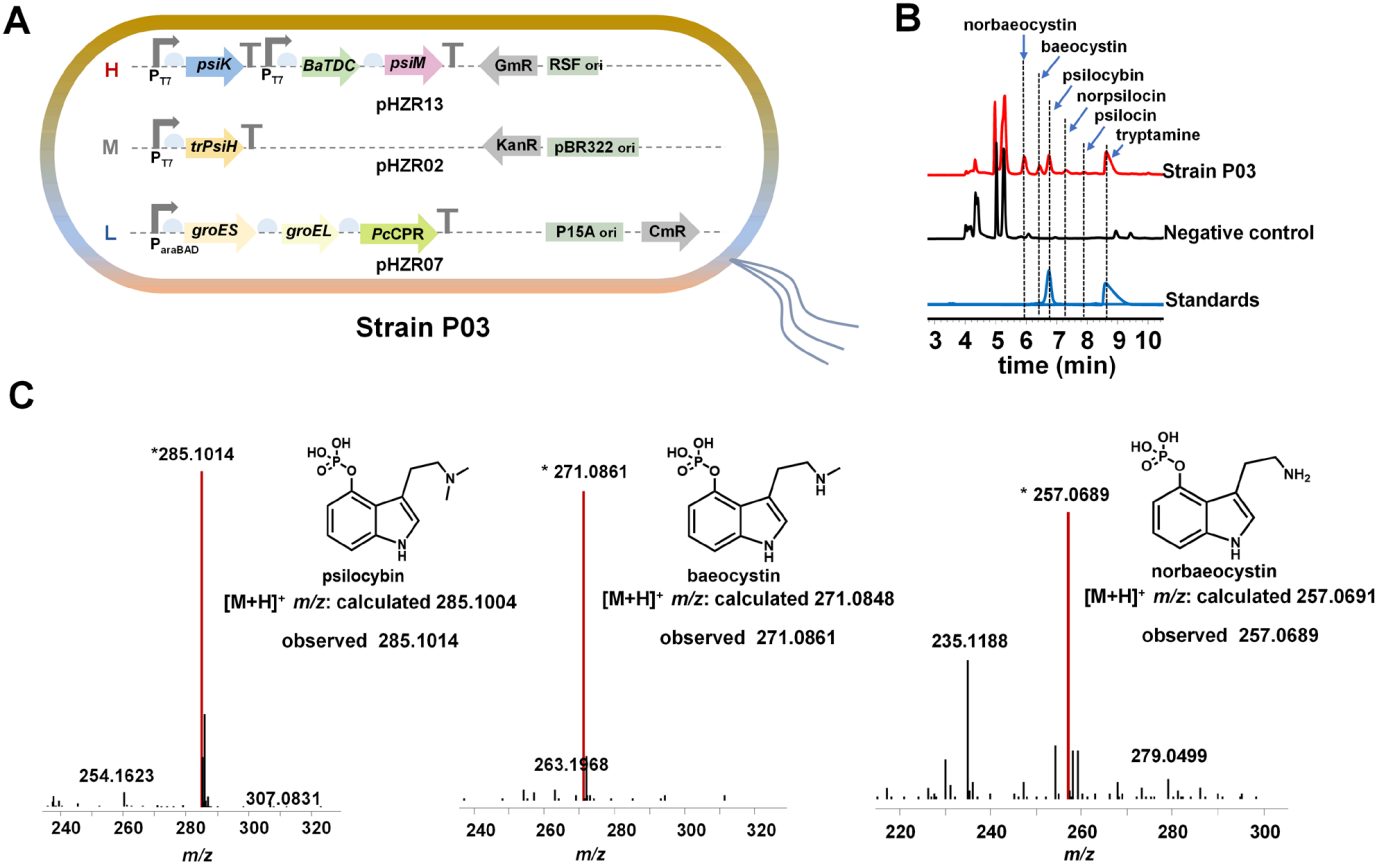
Reconstitution of the entire psilocybin biosynthetic pathway in *Escherichia coli* . (A) Gene architecture for de novo synthesis of psilocybin in strain P03. (B) HPLC analysis of the products produced by strain P03. The extract from the *E. coli* strain carrying the empty plasmids was used as the negative control. (C) HRMS analysis of the products produced by strain P03.

Culture temperature is critical for the enzymatic activities in the de novo biosynthetic pathway of psilocybin. According to the effect of culture temperature on enzymatic performance, we found that low temperature was beneficial for PsiH and BaTDC (Figure S2C and 3B), and relatively high temperature was beneficial for PsiK and PsiM (Figure S3D). Considering that the decarboxylation of tryptophan catalysed by BaTDC is the first step and the hydroxylation of tryptamine catalysed by PsiH is the rate‐limiting step to obtain de novo biosynthesis of psilocybin in *E. coli* , we expressed the whole biosynthetic enzymes at 20°C, which was the appropriate temperature for BaTDC and PsiH expression, to ensure the abundant precursors for psilocybin biosynthesis. To obtain the de novo biosynthesis of psilocybin in *E. coli* , *BaTDC*, *psiK* and *psiM* were cloned into the high copy number plasmid pRSFDuet‐GmR, and the resulting plasmid pHZR13 was co‐transformed into *E. coli* BL21 together with plasmids pHZR02 and pHZR07 (Figure 2A), resulting in the initial strain P03. The strain P03 was cultured at 20°C for 24 h. The products were detected by HPLC analysis. As shown in Figure 2B, psilocybin was produced compared to the negative control and authentic standard. HRMS analysis further showed that the incomplete methylation intermediates norbaeocystin and baeocystin remained (Figure 2B,C, Figure S5). In addition, both norpsilocin and psilocin, the spontaneous or enzyme‐catalysed dephosphorylation products of baeocystin and psilocybin (Milne et al. 2020), were also detected (Figure 2B, Figure S5).

### 3.3 Engineering the Electron Transfer Pathway to Improve Psilocybin Production

**FIGURE 3:**
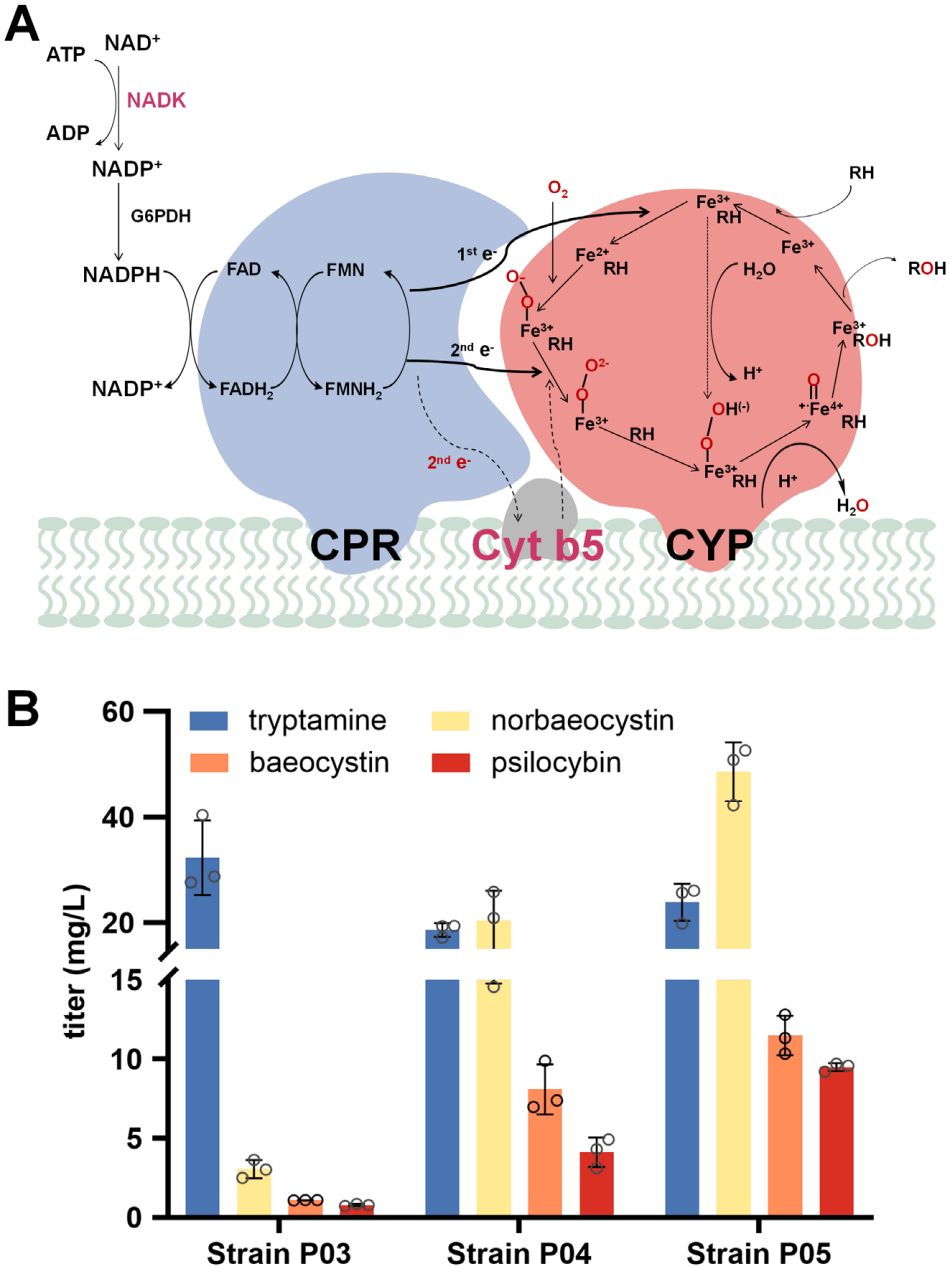
Engineering the electron transfer pathway to enhance psilocybin production in *Escherichia coli* . (A) Schematic representation of the electron transfer pathway of the P450 monooxygenase. (B) The production of tryptamine, norbaeocystin, baeocystin and psilocybin produced by the engineered strains P03, P04 (expressing *PcCYB5*) and P05 (expressing *PcCYB5* and *nadK*).

The successful biosynthesis of psilocybin indicated that *E. coli* could be developed into a robust platform for eukaryotic P450 characterisation and engineering (Park et al. 2022). The biosynthesis of 4‐hydroxytryptamine catalysed by PsiH requires a two‐ET from NADPH. The low titre of psilocybin and a large amount of residual tryptamine in strain P03 prompted us to improve the activity of PsiH by engineering its electron transfer (ET) pathway. PsiH belongs to the fungal class II P450 system (Durairaj et al. 2016), and its ET pathway consists of a membrane‐bound protein CPR containing the cofactors FAD and FMN, which transfer two electrons from NAD(P)H to the heme moiety (Figure 3A). Alternatively, it may contain a third small protein component, cytochrome b5, which transfers a second electron to the heme moiety, and overexpression of *CYB5* to enhance the ET pathway has gained much success in enhancing production (Paddon et al. 2013; Sun et al. 2022; Yao et al. 2022). To improve the ET efficiency, the *PcCYB5* gene from *P. cubensis* was polycistronically expressed with *PcCPR* in P03. The resulting strain P04 did give a significantly increased titre of norbaeocystin, baeocystin and psilocybin at 20.4, 8.1 and 4.1 mg/L, respectively, which was 5.7, 6.5 and 4.7‐fold higher than that of the parental strain P03 (Figure 3B). In addition, the titre of tryptamine in strain P04 decreased to 18.6 mg/L, which was 57.6% lower than that of strain P03 (Figure 3B). These results indicated that the catalytic activity of PsiH was enhanced by improving the ET pathway.

Redox cofactor NAD(P)H is the important electron source in cellular primary and secondary metabolism and is indispensable for the catalytic cycle of fungal P450s. Lack of NAD(P)H could reduce P450 activity due to inefficient electron transfer. Improving cellular NADPH levels could enhance the production of P450‐involved biosynthesis (Liu et al. 2021). Thus, we rerouted redox metabolism to improve the activity of PsiH. Previous studies showed that overexpression of the NAD+ kinase gene (*nadK*) in *E. coli* improved NADPH supply, which further led to bacterial isobutanol production (Shi et al. 2013). Therefore, we overexpressed the native *nadK* gene on the plasmid pCDFDuet‐Amp in the strain P04. The resulted strain P05 gave increased titers of norbaeocystin, baeocystin and psilocybin at 48.6, 11.5 and 9.5 mg/L, which were increased by 134%, 42% and 132% compared to that of its parental strain P04, respectively (Figure 3B), demonstrating that the catalytic activity of PsiH was improved by enhancing NADPH supply.

### 3.4 Increasing Tryptophan Accumulation to Improve Psilocybin Production

**FIGURE 4:**
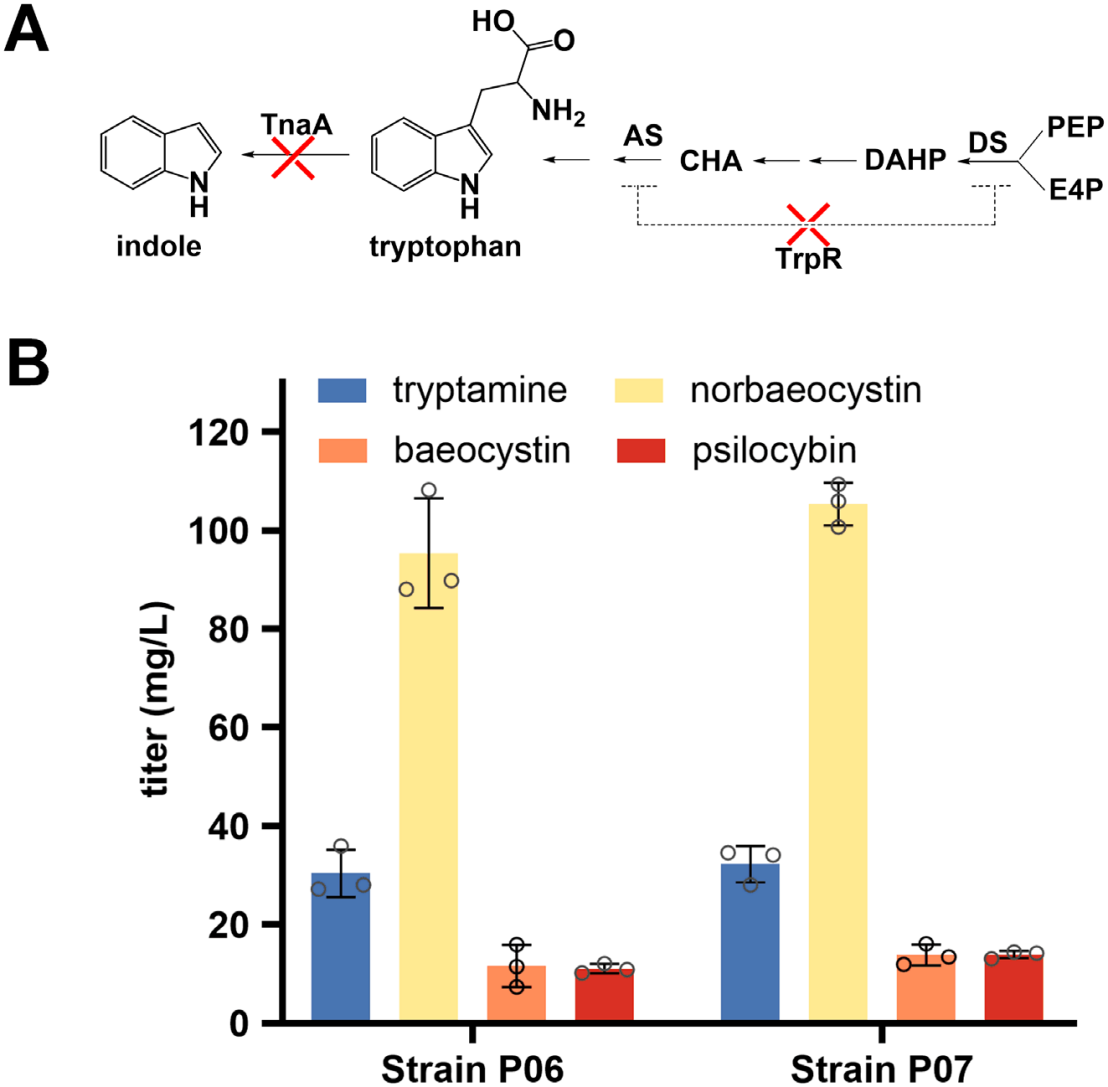
Engineering tryptophan supply to enhance psilocybin production. (A) Schematic illustration of the strategies designed to increase tryptophan supply in *Escherichia coli* . E4P, erythrose 4‐phosphate; PEP, phosphoenolpyruvate; DS, 3‐deoxy‐D‐arabino‐heptulosonate‐7‐phosphate synthase; DAHP, 3‐deoxy‐D‐arabino‐heptulosonate‐7‐phosphate; CHA, chorismic acid; AS, anthranilic acid synthetase; TrpR, tryptophan repressor factor; TnaA, tryptophanase. (B) The production of tryptamine, norbaeocystin, baeocystin and psilocybin produced by the sequentially engineered strains P06 (ΔtnaA) and P07 (ΔtnaAΔtrpR).

Adequate precursor supply is critical for the biosynthesis of target compounds (Sang et al. 2023). Since tryptophan is the starting precursor for psilocybin biosynthesis, increasing tryptophan accumulation would enhance psilocybin production. The biosynthetic pathway of tryptophan has been extensively studied, and there are a number of reports describing modifications for increasing the supply of tryptophan (Ren et al. 2023). Tryptophanase (TnaA) catalyses the conversion of tryptophan to indole (Figure 4A), so knocking out the *tnaA* gene would block the metabolic flow to indole and thus increase tryptophan accumulation (Lee et al. 2020). We obtained the *tnaA* disruption mutant of *E. coli* BL21 by using the CRISPR/Cas9‐mediated gene editing tools (Figure S6) as described previously (Jiang et al. 2015) and named it strain P01. Then, *PcCYB5*, *nadK* and the psilocybin biosynthetic genes were expressed in strain P01, resulting in strain P06. The titers of tryptamine, norbaeocystin, baeocystin and psilocybin were 30.4, 95.4, 11.6 and 11.1 mg/L, which were improved by 27%, 96%, 0% and 17%, respectively, compared with those of strain P05(Figure 3B), showing that blocking the degradation of tryptophan could significantly improve the production of norbaeocystin. But the production of baeocystin and psilocybin was hardly improved, indicating that methylation was the rate‐limiting step. We further relieved the feedback inhibition of tryptophan biosynthesis in strain P01 by knocking out the transcriptional repressor encoding gene *trpR* (Figure 4A, Figure S6), resulting in strain P02. By co‐expressing *PcCYB5*, *nadK* and the psilocybin biosynthetic genes in strain P02, the resulting strain P07 produced tryptamine, norbaeocystin, baeocystin and psilocybin at the titers of 32.3, 105.3, 13.9 and 14.0 mg/L, representing an increase of 6.3%, 10.1%, 19.8% and 26.1%, respectively, over strain P06.

### 3.5 Enhancing Methylation to Improve Psilocybin Production

**FIGURE 5:**
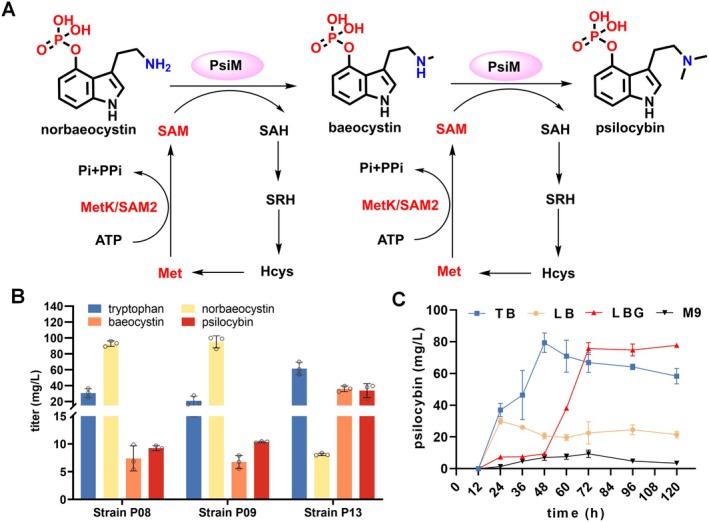
Enhancing methylation to improve psilocybin production. (A) Schematic representation of PsiM‐catalysed sequential methylation reactions. SAM, S‐adenosyl‐L‐methionine; SAH, S‐adenosyl‐L‐homocysteine; SRH, S‐ribosyl‐homocysteine; Hcys, homocysteine; Met, methionine; MetK, methionine adenosyltransferase; SAM2, S‐adenosylmethionine synthetase. (B) The production of tryptamine, norbaeocystin, baeocystin and psilocybin produced by the sequentially engineered strains P08 (expressing *metK*), P09 (expressing *SAM2*) and P13 (expressing an extra copy of *psiM*). (C) The production of psilocybin produced by strain P13 through cultivation in different media.

Psilocybin is a dimethylated compound, and *S*‐adenosyl‐L‐methionine (SAM) acts as the major methyl donor (Figure 5A). In the strain P07, the total titre of norbaeocystin and baeocystin, which were the major components of the products, was 119.2 mg/L. The two possible reasons were that: (i) the lack of SAM supplementation for PsiM‐involved methylation; (ii) the insufficient catalytic ability of methyltransferase PsiM. To test whether the supply of methyl donor was a limiting factor for psilocybin synthesis (Kunjapur et al. 2016; Shen et al. 2022), 0.5 mg/mL methionine (Met) was fed into the culture of strain P07. The results showed that strain P07 produced 11.9 mg/L psilocybin (Figure S7), which was lower than that of the control group. We further activated the SAM cycle by overexpressing the methionine adenosyltransferase gene to regenerate SAM. We first overexpressed the native *metK* gene (Liu et al. 2022) in strain P07 on plasmid pCDFDuet‐Amp, but the resulting strain P08 showed decreased production of baeocystin and psilocybin (Figure 5B). When *metK* was replaced by the *SAM2* gene (Liu et al. 2022) from *S. cerevisiae* (strain P09), we found a similar result (Figure 5B). These results indicated that SAM supply was not the limiting factor for the catalytic efficiency of PsiM.

Since PsiM is the only enzyme that catalyses the successive methylation reaction of converting norbaeocystin to the final product, its catalytic activity is crucial for psilocybin production. To obtain a PsiM enzyme with higher catalytic activity, the cDNA of *PacPsiM* from *Panaelus cyanescens*, *GdPsiM* from *Gymnopilus dilepis* and *PscPsiM* from *Psilocybe cyanescens* (Figure S8) was codon optimised and synthesised to replace *PcPsiM* in strain P07 to generate strains P10–12 (Table 1). However, no strain produced a higher titre of psilocybin than that of strain P07 (Figure S9), suggesting that *PcPsiM* from *P. cubensis* was the optimal allele in *E. coli* .

We further increased an additional copy of *PcPsiM* on plasmid pHZR02 using an independent T7 expression frame after the *psiH* gene. The resulting plasmid pHZR20 was co‐transformed into strain P02 with plasmids pHZR08, pHZR13 and pHZR14 to generate the strain P13. Strain P13 showed an obviously increased psilocybin titre of 33.8 mg/L in flask fermentation, which was 1.4 times higher than that of strain P07. The titre of unmethylated norbaeocystin was 8.1 mg/L, which was 92.3% lower than that of strain P07, demonstrating that increasing the copy number of the *psiM* gene could greatly enhance the first step of methylation. However, excessive amounts of one‐step methylation product baeocystin (36.0 mg/L) were accumulated, indicating that the second round of methyl transfer was still inefficient, which may be attributed to the higher catalytic efficiency of PsiM towards norbaeocystin than that of baeocystin (Hudspeth and Rogge 2024).

### 3.6 Fermentation Medium Optimisation

To test the ability of the engineered strain in de novo synthesising psilocybin, a modified M9 medium was used for fermentation. The result showed that psilocybin reached its maximum titre of 9.4 mg/L at 72 h, demonstrating that psilocybin was de novo synthesised in strain P13. However, the production was lower compared to the previous *E. coli* co‐culture strategy (Flower et al. 2023), which may be attributed to the poor growth of strain P13 in M9 medium. To optimise the culture condition for improving psilocybin production, strain P13 was cultured in different fermentation media. Strain P13 was subjected to 100 mL of LB, LBG and TB medium, respectively, and was cultured in 500 mL baffled fermentation flasks. The results showed that psilocybin reached its maximum titre of 29.9 mg/L at 24 h in LB medium, 79.4 mg/L at 48 h in TB medium and 75.7 mg/L at 72 h in LBG medium, respectively (Figure 5C). The highest psilocybin production in TB medium increased by 232.2% compared to LB medium and was a 100‐fold improvement over the starting strain P03. LBG medium also produced a high titre of psilocybin, but it required a longer culture time than that of TB medium (Figure 5C), which may be attributed to the fact that *E. coli* consumed glucose for rapid growth when LBG medium was used, and the accumulation of acetic acid inhibited product synthesis until glucose was depleted (Lee 1996). Whereas the carbon source of TB is glycerol, *E. coli* consumed glycerol at a slow rate, resulting in a decrease in the carbon flux entering the fermentation and less accumulation of acetic acid (Lee and Chang 1990). Therefore, our results suggest that TB medium is suitable for psilocybin production in flask fermentation.

## 4 Discussion

In this study, de novo biosynthesis of psilocybin was successfully achieved in a single *E. coli* host. Due to the membrane‐bound nature of the P450 enzyme PsiH and the need for enzymatic redox partners for catalysis, de novo synthesis of psilocybin in *E. coli* has been hindered (Adams et al. 2019; Flower et al. 2023). The N‐terminal domains of most eukaryotic cytochrome P450 enzymes contain membrane‐anchoring regions that are intrinsically embedded within the endoplasmic reticulum or mitochondrial membrane structures (Durairaj and Li 2022). This frequently leads to the formation of substantial insoluble protein aggregates, significantly impeding their expression in *E. coli* (Hausjell et al. 2018). Thus, we expressed PsiH together with the molecular chaperone proteins such as GroES/GroEL, which could facilitate the proper folding of eukaryotic P450s to obtain the active enzymes (Ahn et al. 2004; Ichinose et al. 2015). Previous studies have shown that N‐terminal modifications could improve the expression of eukaryotic P450s in *E. coli* , such as deleting the hydrophobic domain (Park et al. 2014) or replacing the hydrophobic domain with the N‐terminal sequence of other P450s (Ichinose et al. 2015; Ichinose and Wariishi 2013). In this study, many PsiH variants with N‐terminal hydrophobic domain modifications were constructed (Figure 1A). We found that deleting the N‐terminal hydrophobic domain of PsiH could obviously increase its expression level (Figure 1B), such as the variants trPsiH, SUMO‐trPsiH and SUMO‐5144C1NTD‐trPsiH. By co‐expressing the redox partner PcCPR from *P. cubensis* , all constructed PsiH variants showed the tryptamine hydroxylase activity, and the production of 4‐hydroxytryptamine was positively related to the expression level of the chimeric PsiH proteins, especially as the strain H2 expressing *trPsiH* produced the highest titre of 4‐hydroxytryptamine at 107.82 mg/L, which was 10.53‐fold higher than that of strain H1 expressing the wild‐type *psiH* (Figure 1C). The engineered PsiH showed improved catalytic activity. Using the bioactive trPsiH, the entire biosynthetic pathway of psilocybin was successfully reconstituted, and psilocybin was de novo synthesised in *E. coli* .

Since the PsiH‐catalysed hydroxylation is the rate‐limiting step in psilocybin biosynthesis, PsiH activity is critical for high production. In addition to the N‐terminus modifications of PsiH, we further engineered its ET pathway to improve PsiH catalytic activity. CPR sequentially transfers two electrons from NAD(P)H to activate molecular oxygen in P450‐mediated reactions and plays a key role in the ET pathway (Durairaj et al. 2016; Durairaj and Li 2022). In addition to CPR, a third protein component, CYB5, can assist in the second ET (Durairaj et al. 2016; Durairaj and Li 2022), and overexpression of CYB5 to enhance the ET pathway has gained much success in other works (Paddon et al. 2013; Sun et al. 2022; Yao et al. 2022). By overexpressing PcCYB5, the titre of psilocybin in strain P04 was greatly improved by 4.7‐fold compared to that of the parental strain P03. We then increased the efficiency of the ET pathway by improving the supply of NADPH, which further increased psilocybin production by 132%.

To obtain de novo biosynthesis of psilocybin in a single cell, the biosynthetic enzymes must be expressed under a concordant conditions, especially the expression temperature. PsiH and BaTDC showed their best conversion activities at 20°C (Figures S2C and S3B), and the catalytic activity of PsiH decreased sharply when the expression temperature was higher than 25°C, while PsiK and PsiM gave increased titers of psilocybin with the increase of culture temperature from 18°C to 37°C (Figure S4B). Since BaTDC and PsiH catalyse the early process of psilocybin biosynthesis, we expressed the whole biosynthetic enzymes at 20°C to ensure the abundant precursors for psilocybin biosynthesis. Based on this temperature, psilocybin was successfully de novo synthesised in *E. coli* . Finally, by optimising the flask fermentation conditions, 79.4 mg/L of psilocybin was produced in the TB medium without the addition of substrates such as 4‐hydroxyindole, serine and methionine.

In order to efficiently reconstitute the psilocybin biosynthetic pathway in *E. coli* , its biosynthetic genes were carefully expressed using the plasmids with different copy numbers, as psilocybin production was sensitive to the expression level of pathway genes (Adams et al. 2019). First, the key gene *psiH* was expressed based on the medium‐copy number plasmid pET28a to ensure sufficient PsiH for catalysis in the rate‐limiting step and to prevent superfluous accumulation of PsiH inclusion for its inhibitory effect on cell growth. Second, the ET pathway proteins were expressed based on the low‐copy number plasmid pGro7. The ET pathway is essential for the catalytic ability of eukaryotic P450s, but CPRs and CYB5 are both membrane‐bound proteins that would cause a severe endoplasmic reticulum (ER) stress when they are overexpressed in the cell (Hu et al. 2022). Generally, there is only one or two CPR genes in a fungal genome that could coordinate many P450s (Durairaj et al. 2016), and a high P450:CPR ratio was beneficial for improving the catalytic activity of P450s (Sun et al. 2023). Thus, the expression of *PcCPR* and *PcCYB5* genes based on the low‐copy number plasmid pGro7 greatly improved the production of psilocybin (Figure 3). Third, and most importantly, *BaTDC*, *psiK* and *psiM* genes were expressed based on the high‐copy number plasmid pRSFDuet, which resulted in high psilocybin production in the engineering strain.

Psilocybin has two methyl groups that are transferred from SAM catalysed by the methyltransferase PsiM (Figure 5A). The accumulation of norbaeocystin and baeocystin in strain P07 (Figure 4B) indicated that the methylation step was impaired. Increasing the supply of SAM by adding methionine in the culture (Figure S7), or overexpressing different methionine adenosyltransferase genes did not increase psilocybin production (Figure 5B), indicating that SAM was not the limiting factor for the PsiM catalytic activity. Increasing PsiM expression by adding an extra copy of the *psiM* gene improved psilocybin production in strain P13 by 1.4‐fold and decreased the accumulation of the unmethylated intermediate norbaeocystin by 92.3% (Figure 5B), indicating that the first methylation step was greatly enhanced, which further improved psilocybin production (Milne et al. 2020). However, excessive amounts of baeocystin were accumulated in strain P13, which may be attributed to the lower affinity of PsiM for baeocystin compared to that for norbaeocystin, and a twofold Kcat value of the first methyl transfer compared to the second methylation reaction (Hudspeth and Rogge 2024). Thus, to further improve psilocybin production, more efforts are needed for PsiM engineering to increase the efficiency of the second methylation reaction.

## 5 Conclusion

In conclusion, we have established an *E. coli* ‐based platform capable of de novo producing psilocybin without any precursor feeding. Eukaryotic P450s are generally thought to perform poorly in prokaryotic cells, but through the N‐terminus modification and ET pathway optimisation, our work provides an excellent example of engineering eukaryotic P450s to achieve higher catalytic efficiency and synthesise naturally valuable products in a prokaryotic host. Combined with strategies of engineering the precursor supply and enhancing the final methylation reaction, psilocybin was effectively de novo synthesised in a single *E. coli* cell for the first time. Our work demonstrates the complete production of psilocybin in a prokaryotic host and provides a sustainable route for psilocybin to fulfil the industrial production and pharmaceutical application.

## Author Contributions

**Zhangrao Huang:** data curation, investigation, visualization, writing – original draft. **Yongpeng Yao:** conceptualization, data curation, investigation, supervision, visualization, funding acquisition, writing – original draft, writing – review and editing. **Rouyu Di:** investigation. **JianChao Zhang:** investigation. **Yuanyuan Pan:** investigation. **Gang Liu:** conceptualization, supervision, funding acquisition, project administration, resources, writing – review and editing.

## Ethics Statement

This study does not contain any studies with human participants or animals performed by any of the authors.

## Conflicts of Interest

Zhangrao Huang, Yongpeng Yao, Yuanyuan Pan and Gang Liu are inventors on a provisional patent application related to this work (no. CN202410238362.8, filed 3 March 2024). All other authors declare that they have no competing interests.

## Supporting information


Data S1:

## Data Availability

The data supporting the findings of this study are available in the manuscript and its Supporting Information S1.
