# Supplementary material for: De Novo Biosynthesis of Antidepressant Psilocybin in Escherichia coli
Source: Microb Biotechnol. 2025 Apr 3;18(4):e70135. doi: 10.1111/1751-7915.70135 (PMC11966237; doi:10.1111/1751-7915.70135)
Supplement: Supplementary file 1 — Data S1: [file MBT2-18-e70135-s001.docx]

SUPPORTING INFORMATION

**De novo biosynthesis of antidepressant psilocybin in *Escherichia coli***

Zhangrao Huang^1, 2^ **·** Yongpeng Yao^1,*^ **·** Rouyu Di^1, 2^ **·** JianChao Zhang^1^ **·** Yuanyuan Pan^1^ **·** Gang Liu^1, 2,*^

^1^State Key Laboratory of Mycology, Institute of Microbiology, Chinese Academy of Sciences, Beijing 100101, P.R. China

^2^University of Chinese Academy of Sciences, Beijing 100049, P.R. China

Zhangrao Huang and Yongpeng Yao contributed equally to this work.

*Corresponding author.

Gang Liu

E-mail: [liug@im.ac.cn](mailto:liug@im.ac.cn)

Yongpeng Yao

E-mail: [yaoyp@im.ac.cn](mailto:yaoyp@im.ac.cn)

Content

Table S1 The plasmids used in this study

Table S2 The primers used in this study

Table S3 The proteins expressed in this study

Table S4 The codon-optimized sequences expressed in this study

Figure S1 Prediction of the transmembrane region of PsiH using TMHMM v2.0

Figure S2 The catalytic ability of PsiH variants

Figure S3 The catalytic ability of different tryptophan decarboxylases

Figure S4 The catalytic ability of PsiK and PsiM

Figure S5 LC-HRMS analysis of products produced by strain P03

Figure S6 PCR validation of the gene knockout *E. coli* strains

Figure S7 The titers of psilocybin produced in the strain P07 with or without feeding methionine

Figure S8 Mining of PsiM homologues

Figure S9 The titers of psilocybin produced in the strains expressing different PsiM homologues

Figure S10 Summary of the standard curves

Supplementary Tables

Table S1 The plasmids used in this study

| **Plasmid** | **Description** | **Backbone** | **Reference** |
| --- | --- | --- | --- |
| pET28a | Vector backbone, containing *pBR322 ori*, *P_T7_*, *Nde*I/*Xho*I restriction site, *kana* cassette | pET28a | Novagen |
| pGro7 | Vector backbone, containing *groES*, *groEL*, *P_ara_*, *p15A ori*, *chl* cassette | pGro7 | Novagen |
| pCDFDuet-1 | Vector backbone, containing *P_T7_*, *BamH*I/*Not*I restriction site, *CloDF13 ori*, *str* cassette | pCDFDuet | Novagen |
| pRSFDuet-1 | Vector backbone, containing *P_T7_*, *RSF ori*, *kana* cassette | pRSFDuet | Novagen |
| pCDFDuet-Amp | Vector backbone, containing *P_T7_*, *BamH*I/*Not*I restriction site, *CloDF13 ori*, *ampR* cassette | pCDFDuet-1 | This study |
| pRSFDuet-GmR | Vector backbone, containing *P_T7_*, *RSF ori*, *gmR* cassette | pRSFDuet-1 | This study |
| pHZR01 | *P_T7_::psiH* | pET28a | This study |
| pHZR02 | *P_T7_::trpsiH* | pET28a | This study |
| pHZR03 | *P_T7_::5144C1NTD-trpsiH* | pET28a | This study |
| pHZR04 | *P_T7_::SUMO-trpsiH* | pET28a | This study |
| pHZR05 | *P_T7_::SUMO-psiH* | pET28a | This study |
| pHZR06 | *P_T7_::SUMO-5144C1NTD-trpsiH* | pET28a | This study |
| pHZR07 | *P_ara_::PcCPR* | pGro7 | This study |
| pHZR08 | *P_ara_:: PcCPR+PcCYB5* | pGro7 | This study |
| pHZR09 | *P_T7_:: psiD* | pRSFDuet-GmR | This study |
| pHZR10 | *P_T7_:: BaTDC* | pRSFDuet-GmR | This study |
| pHZR11 | *P_T7_:: CrTDC* | pRSFDuet-GmR | This study |
| pHZR12 | *P_T7_:: psiK+psiM* | pRSFDuet-GmR | This study |
| pHZR13 | *P_T7_:: psiK+BaTDC+psiM* | pRSFDuet-GmR | This study |
| pHZR14 | *P_T7_::nadK* | pCDFDuet-Amp | This study |
| pHZR15 | *P_T7_::metK-nadK* | pCDFDuet-Amp | This study |
| pHZR16 | *P_T7_::SAM2-nadK* | pCDFDuet-Amp | This study |
| pHZR17 | *P_T7_:: psiK+BaTDC+PacPsiM* | pRSFDuet-GmR | This study |
| pHZR18 | *P_T7_:: psiK+BaTDC+GdPsiM* | pRSFDuet-GmR | This study |
| pHZR19 | *P_T7_:: psiK+BaTDC+PscPsiM* | pRSFDuet-GmR | This study |
| pHZR20 | *P_T7_::trpsiH+psiM* | pET28a | This study |
| pTarget-trpR-2 | *trpR-*sgRNA | pTarget | This study |
| pTarget-trpR-1 | *trpR-*sgRNA | pTarget | This study |
| pTarget-tnaA-2 | *tnaA*-sgRNA | pTarget | This study |
| pTarget-tnaA-1 | *tnaA*-sgRNA | pTarget | This study |
| pCas-prha-NEW | KanR, rep101 ori, Cas9 protein, λ-Red recombinase under araBAD promoter | pCas | ([Jiang et al. 2015](#_ENREF_1)) |
| pTarget | sgDNA |  | ([Jiang et al. 2015](#_ENREF_1)) |

Table S2 The primers used in this study

| **Primer** | **Sequence (5’-3’)** | **Function** |
| --- | --- | --- |
| 28a-psiH-F | CGGCCTGGTGCCGCGCGGCAGCCATATGATCGCCGTCCTGTTCAGC | For *psiH* amplification |
| 28a-psiH-R | GGTGGTGGTGGTGGTGCTCGAGCTAAAGCTTTGGACCGGACACGCT |  |
| 28a-SUMO-F | CGGCCTGGTGCCGCGCGGCAGCCATATGAGTGATAGCGAAGTTAAT | For *SUMO* amplification |
| 28a-SUMO-R | GCCACCAATCTGTTCGCGATG |  |
| 28a-SUMO-psiH-F | AGCACATCGCGAACAGATTGGTGGCATGATCGCCGTCCTGTTCAGC | For *psiH* amplification |
| 28a-SUMO-psiH-R | GGTGGTGGTGGTGGTGCTCGAGCTAAAGCTTTGGACCGGACACGCT |  |
| 28a-trPsiH-F | CGGCCTGGTGCCGCGCGGCAGCCATATGCCACCTGGTCCACCAGGTAT | For *trpsiH* amplification |
| 28a-trPsiH-R | GGTGGTGGTGGTGGTGCTCGAGCTAAAGCTTTGGACCGGACACGCT |  |
| 5144C1NTD-F | CCTGGTGCCGCGCGGCAGCCATATGTCTCTGCTGCTCGCCGCCACGCTGTTCCTTCACT | For *5144C1NTD* amplification |
| 5144C1NTD-R | TACCTGGTGGACCAGGTGGGAGCGGATAACGCTTCTGCCTGGAGTGAAGGAACAGCGTG |  |
| 28a-5144-trPsiH-F | CTCCCACCTGGTCCACCAGG | For *trpsiH* amplification |
| 28a-5144-trPsiH-R | GGTGGTGGTGGTGGTGCTCGAGCTAAAGCTTTGGACCGGACACGCT |  |
| 28a-SUMO-5144-F | AGCACATCGCGAACAGATTGGTGGCATGTCTCTGCTGCTCGCCGCCACGCTGTTCCTTCACT | For *5144C1NTD* amplification |
| 28a-SUMO-5144-R | TACCTGGTGGACCAGGTGGGAGCGGATAACGCTTCTGCCTGGAGTGAAGGAACAGCGTG |  |
| 28a-SUMO-trPsiH-F | AGCACATCGCGAACAGATTGGTGGCCTCCCACCTGGTCCACCAG | For *trpsiH* amplification |
| 28a-SUMO-trPsiH-R | GGTGGTGGTGGTGGTGCTCGAGCTAAAGCTTTGGACCGGACACGCT |  |
| RSF-PsiK-F | TAACTTTAATAAGGAGATATACCATGGCCTTCGACCTCAAGAC | For *psiK* amplification |
| RSF-PsiK-R | CTTAAGCATTATGCGGCCGCCTAAAGCTTGGCGGTGCTGGAC |  |
| RSF-PsiD-F | AAGTATAAGAAGGAGATATACATATGCAGGTCATCCCTGCATGC | For *psiD* amplification |
| RSF-PsiD-R | AAGTATAAGAAGGAGATATACATATGCAGGTCATCCCTGCATGC |  |
| RSF-BaTDC-F | AAGTATAAGAAGGAGATATACATATGAGCGAAAACCTGCAGCTGA | For *BaTDC* amplification |
| RSF-BaTDC-R | CATTGATAACTCTCCTTTGAGAACTATTCTGCAACGCAAGGATAGCTAA |  |
| RSF-CrTDC-F | AAGTATAAGAAGGAGATATACATATGGGTAGCATTGATAGCACGAAC | For *CrTDC* amplification |
| RSF-CrTDC-R | ATTGATAACTCTCCTTTGAGAACTACGCTTCTTTCAGCAGATCATC |  |
| RSF-PsiM-F | AGTTCTCAAAGGAGAGTTATCAATGCACATCCGCAACCCATAC | For *psiM* amplification |
| RSF-PsiM-R | AGCGGTTTCTTTACCAGACTCGAGTTAAAGCTTGAAGAGGCTGGACAG |  |
| RSF-1-F | CTCGAGTCTGGTAAAGAAACCG | For pRSFDuet-GmR vector  backbone amplification |
| RSF-1-R | ATGTATATCTCCTTCTTATACTTAACTAATATACTAAG |  |
| RSF-2-F | AGGCGGCCGCATAATGCTTAAG | For pRSFDuet-GmR vector  backbone amplification |
| RSF-2-R | GGTATATCTCCTTATTAAAGTTAAACAAAATTATTTCTACAGGGGA |  |
| pGro7-CPR-F | GTAAAGTATAAGAAGGAGATATACATATGATGGCAAGCAGCAGCAGCGA | For *CPR* amplification |
| pGro7-CPR-R | TTATTTCTGCGAGGTGCAGGGCAATTAGCTCCAAACATCCAGCATCAG |  |
| pGro7-CYB5-F | GTATCTGAAAGGGGATACGCATGAGCGCAGATAAAATTGT | For *CYB5* amplification |
| pGro7-CYB5-R | TTATTTCTGCGAGGTGCAGGGCAATCATTATGCACTACCTGAATAATAC |  |
| pGro7-F | TTGCCCTGCACCTCGCAGAAATAA | For pGro7 vector  backbone amplification |
| pGro7-R | CATATGTATATCTCCTTCTTATACTTTACATCATGCCGCCCATGCC |  |
| RSF-1-F | CTCGAGTCTGGTAAAGAAACCG | For pRSFDuet-GmR vector  backbone amplification |
| RSF-1-R | ATGTATATCTCCTTCTTATACTTAACTAATATACTAAG |  |
| RSF-2-F | AGGCGGCCGCATAATGCTTAAG | For pRSFDuet-GmR vector  backbone amplification |
| RSF-2-R | GGTATATCTCCTTATTAAAGTTAAACAAAATTATTTCTACAGGGGA |  |
| pGro7-CPR-F | GTAAAGTATAAGAAGGAGATATACATATGATGGCAAGCAGCAGCAGCGA | For *CPR* amplification |
| pGro7-CPR-R | TTATTTCTGCGAGGTGCAGGGCAATTAGCTCCAAACATCCAGCATCAG |  |
| pGro7-CYB5-F | GTATCTGAAAGGGGATACGCATGAGCGCAGATAAAATTGT | For *CYB5* amplification |
| pGro7-CYB5-R | TTATTTCTGCGAGGTGCAGGGCAATCATTATGCACTACCTGAATAATAC |  |
| pGro7-F | TTGCCCTGCACCTCGCAGAAATAA | For pGro7 vector  backbone amplification |
| pGro7-R | CATATGTATATCTCCTTCTTATACTTTACATCATGCCGCCCATGCC |  |
| CDF-nadK-F | ACTTTAATAAGGAGATATACCATGAATAATCATTTCAAGTGTATTGGC | For *nadK* amplification |
| CDF-nadK-R | TTGTCGACCTGCAGGCGCGCCGAGTTAGAATAATTTTTTTGACCAGCCG |  |
| T7-PsiM-F | ATCCGGATATAGTTCCTCCTTTCAG | For *psiM* amplification |
| T7-PsiM-R | TAATACGACTCACTATAGGGGAATT |  |
| 28a-trpsiH-F | AATTCCCCTATAGTGAGTCGTATTACAAAAAACCCCTCAAGACCC | For 28a-*trpsiH* amplification |
| 28a-trpsiH-R | CTGAAAGGAGGAACTATATCCGGAT |  |
| H-test-F | CTTTGTTAGCAGCCGGATC | For the verification of psiH chimerism |
| H-test-R | GGCGTAGAGGATCGAGATCT |  |
| UP-tnaA-F | GCTTCGCTTCATTGTTAGCA | For donor DNA- tnaA amplification |
| UP-tnaA-R | TACATAATCCTTCATTTATTTTAATTACAGTGATC |  |
| DOWN-tnaA-F | ATTAAAATAAATGAAGGATTATGTATTAATACTACAGAGTGGCTATAAGG | For donor DNA- tnaA amplification |
| DOWN-tnaA-R | CGAACTTATCCACAATACGCTGGCAACGAAAATGGCTGTGCAGATCCCGA |  |
| UP-trpR-F | GCGGTATGCTTTCAACAAC | For donor DNA-trpR amplification |
| UP-trpR-R | AGCGGGGGAAGCAAAATGCCTCAGTAACGACGTCCCCATTCCG |  |
| DOWN-trpR-F | CGGAATGGGGACGTCGTTACTGACTACAAATGCCTGATATACGGCATTGTGAAACGGACT | For donor DNA-trpR amplification |
| DOWN-trpR-R | TCCGTATACTGCTGTAACGTTAA |  |
| sgRNA1-tnaA-F | TAAAGTCGCCGGTATCCGTGGTTTTAGAGCTAGAAATAGCAAGTTAAAAT | For sgDNA amplification |
| sgRNA1-tnaA-R | AAGCTTCTGCAGGTCGACTCTAGAGAATTCAAAAAAAGCACCGACTCGGT |  |
| sgRNA2-tnaA-F | ATCACCAGTAACTCTGCAGGGTTTTAGAGCTAGAAATAGCAAGTTAAAAT | For sgDNA amplification |
| sgRNA2-tnaA-R | AAGCTTCTGCAGGTCGACTCTAGAGAATTCAAAAAAAGCACCGACTCGGT |  |
| sgRNA1-trpR-F | TCTGCCATCGCTGCTGAATAGTTTTAGAGCTAGAAATAGCAAGTTAAAAT | For sgDNA amplification |
| sgRNA1-trpR-R | ATAACAGGGTAATAGATCTAAGCTTCTGCAGGTCGACTCTAGAGAATT |  |
| pTarget-trpR1-R | TATTCAGCAGCGATGGCAGAACTAGTATTATACCTAGGACTGAGCTAGCT | For pTarget vector backbone amplification |
| pTarget-trpR2-R | TTGAGCTGCGCCAGTGGCTGACTAGTATTATACCTAGGACTGAGCTAGCT |  |
| pTarget-tnaA1-R | CACGGATACCGGCGACTTTAACTAGTATTATACCTAGGACTGAGCTAGCT | For pTarget vector backbone amplification |
| pTarget-tnaA2-R | CCTGCAGAGTTACTGGTGATACTAGTATTATACCTAGGACTGAGCTAGCT |  |
| pET28a-test-F | CTTTGGACCGGACACGCT | For verification of the pET28a derived plasmid |
| pET28a-test-R | CTGGTCCACCAGGTATCCCT |  |
| pGro7-test-F | ATGGCAGCTAAAGACGTAAA | For verification of the pGro7 derived plasmid |
| pGro7-test-R | TTACATCATGCCGCCCATGCCA |  |
| pCDFDuet-test-F | GCGACCGAGTGAGCTAGCTA | For verification of the pCDFDuet derived plasmid |
| pCDFDuet-test-R | CAATGCTTAATCAGTGAGGC |  |
| pRSFDuet-test-F | TATGAGCGAAAACCTGCAGC | For verification of the pRSFDuet derived plasmid |
| pRSFDuet-test-R | TTCTGCAACGCAAGGATAGC |  |
| H-test-F | AAGCTTTGGACCGGACACGCT | For verification of the PsiH chimeras |
| H-test-R | TCATCACAGCAGCGGCCTGG |  |
| Cas-test-F | CAATAGGCTTAGATATCGGC | For verification of pCas-prha-NEW |
| Cas-test-R | TTGGACTATCAAAACCACCAT |  |
| tnaA-test-F | GTAAGTAACCGCGCTTACGA | For verification of ΔtnaA |
| tnaA-test-R | GTAAGTAACCGCGCTTACGA |  |
| trpR-test-F | TGGTGAAGAGCAAGTCAAAA | For verification of ΔtrpR |
| trpR-test-R | GGCGGACTAGGTATACCTGT |  |

Table S3 The proteins expressed in this study

| **Protein** | **Function** | **Organism** | **Accession number** |
| --- | --- | --- | --- |
| PsiD | L-tryptophan decarboxylase | *Psilocybe cubensis* | [P0DPA6.1](https://www.ncbi.nlm.nih.gov/protein/P0DPA6.1?report=genbank&log$=protalign&blast_rank=1&RID=GK45YZ83013) |
| PsiH | Tryptamine 4-monooxygenase | *Psilocybe cubensis* | [P0DPA7.1](https://www.ncbi.nlm.nih.gov/protein/P0DPA7.1?report=genbank&log$=protalign&blast_rank=1&RID=GK3R1AFA013) |
| PsiK | 4-hydroxytryptamine kinase | *Psilocybe cubensis* | [P0DPA8.1](https://www.ncbi.nlm.nih.gov/protein/P0DPA8.1?report=genbank&log$=protalign&blast_rank=2&RID=GK495BPT016) |
| PsiM | Methyltransferase | *Psilocybe cubensis* | [P0DPA9.1](https://www.ncbi.nlm.nih.gov/protein/P0DPA9.1?report=genbank&log$=protalign&blast_rank=1&RID=GK4DWCJ3016) |
| PcCPR | Cytochrome P450 reductase | *Psilocybe cubensis* | [XP_047748172.1](https://www.ncbi.nlm.nih.gov/protein/XP_047748172.1?report=genbank&log$=protalign&blast_rank=1&RID=HYYWS7KW013) |
| PcCYB5 | Cytochrome b5 | *Psilocybe cubensis* | [XP_047749914.1](https://www.ncbi.nlm.nih.gov/protein/XP_047749914.1?report=genbank&log$=protalign&blast_rank=1&RID=GK4TJU31013) |
| NadK | NAD+ kinase | *Escherichia coli* | [WP_248420607.1](https://www.ncbi.nlm.nih.gov/protein/WP_248420607.1?report=genbank&log$=protalign&blast_rank=1&RID=GK4ZAHXP016) |
| MetK | *S*-adenosylmethionine synthetase | *Escherichia coli* | AAN81976.1 |
| SAM2 | Methionine adenosyltransferase | *Saccharomyces cerevisiae* | NP_010790.3 |
| BaTDC | Decarboxylase | *Bacillus atrophaeus* | [WP_003327896.1](https://www.ncbi.nlm.nih.gov/protein/WP_003327896.1?report=genbank&log$=protalign&blast_rank=1&RID=VH7FC0W4013) |
| CrTDC | Decarboxylase | *Catharanthus roseus* | [P17770.1](https://www.ncbi.nlm.nih.gov/protein/P17770.1?report=genbank&log$=protalign&blast_rank=1&RID=VH7V76CF016) |
| PacPsiM | Methyltransferase | *Panaelus cyanescens* | PPQ80976.1 |
| GdPsiM | Methyltransferase | *Gymnopilus dilepis* | PPQ70884.1 |
| PscPsiM | Methyltransferase | *Psilocybe cyanescens* | PPQ83230.1 |
| GroEL | Chaperonin | *Enterobacteriaceae* | WP_000729117 |
| GroES | Co-chaperone | *Gammaproteobacteria* | [WP_001026276.1](https://www.ncbi.nlm.nih.gov/protein/WP_001026276.1?report=genbank&log$=protalign&blast_rank=1&RID=VH9BYCGM016) |

Table S4 The codon-optimized sequences expressed in this study

| Genes | Sequences |
| --- | --- |
| *psiH* | ATGATCGCCGTCCTGTTCAGCTTCGTGATCGCCGGTTGCATCTACTACATCGTCAGCCGACGAGTGCGACGATCCCGACTCCCACCTGGTCCACCAGGTATCCCTATCCCATTCATCGGTAACATGTTCGACATGCCTGAGGAGTCCCCATGGCTCACCTTCCTGCAGTGGGGCCGCGACTACAACACCGATATCCTGTACGTGGACGCCGGCGGTACCGAGATGGTCATCCTCAACACCCTGGAGACCATCACCGATCTCCTGGAGAAGCGCGGTAGCATCTACTCCGGCCGCCTCGAGAGCACCATGGTCAACGAGCTGATGGGTTGGGAGTTCGACCTCGGTTTCATCACCTACGGTGATCGATGGCGAGAGGAGCGACGAATGTTCGCAAAGGAGTTCTCCGAGAAGGGCATCAAGCAGTTCCGACACGCACAGGTGAAGGCAGCACATCAGCTCGTCCAGCAGCTGACCAAGACCCCAGACCGATGGGCACAGCACATCCGACATCAGATCGCCGCCATGAGCCTGGACATCGGTTACGGCATCGATCTCGCAGAGGACGATCCATGGCTCGAGGCAACCCACCTGGCAAACGAGGGTCTCGCAATCGCATCCGTCCCTGGCAAGTTCTGGGTGGATAGCTTCCCATCCCTCAAGTACCTGCCAGCATGGTTCCCTGGTGCAGTGTTCAAGCGAAAGGCAAAGGTCTGGCGAGAGGCAGCCGACCATATGGTGGATATGCCCTACGAGACCATGCGCAAGCTGGCACCACAGGGTCTCACCCGACCAAGCTACGCATCCGCACGACTGCAGGCAATGGACCTCAACGGCGATCTGGAGCACCAGGAGCATGTCATCAAGAACACCGCAGCAGAGGTCAACGTGGGTGGTGGTGACACCACCGTGAGCGCAATGTCCGCATTCATCCTCGCCATGGTCAAGTACCCTGAGGTCCAGCGCAAGGTGCAGGCCGAGCTCGATGCCCTGACCAACAACGGTCAGATCCCCGACTACGATGAGGAGGACGATAGCCTCCCTTACCTGACCGCCTGCATCAAGGAGCTGTTCCGCTGGAACCAGATCGCCCCACTGGCCATCCCCCACAAGCTCATGAAGGACGATGTGTACCGCGGCTACCTGATCCCAAAGAACACCCTCGTCTTCGCCAACACCTGGGCCGTCCTGAACGACCCAGAGGTGTACCCAGATCCTTCCGTCTTCCGACCAGAGCGATACCTCGGTCCTGACGGCAAGCCAGATAACACCGTCCGAGACCCACGAAAGGCAGCATTCGGTTACGGTCGACGAAACTGCCCTGGTATCCACCTCGCACAGAGCACCGTGTGGATCGCAGGTGCCACCCTCCTGTCCGCCTTCAACATCGAGCGCCCTGTCGATCAGAACGGCAAGCCCATCGACATCCCTGCCGATTTCACCACCGGCTTCTTCCGCCATCCAGTGCCCTTCCAGTGCCGCTTCGTCCCTCGCACCGAGCAGGTCTCCCAGAGCGTGTCCGGTCCAAAGCTTTAG |
| *psiD* | ATGCAGGTCATCCCTGCATGCAACTCCGCAGCAATCCGAAGCCTCTGCCCAACCCCCGAGTCCTTCCGCAACATGGGCTGGCTGTCCGTCAGCGATGCCGTGTACAGCGAGTTCATCGGCGAGCTCGCAACCCGAGCATCCAACCGAAACTACAGCAACGAGTTCGGCCTGATGCAGCCTATCCAGGAGTTCAAGGCCTTCATCGAGTCCGACCCAGTCGTGCACCAGGAGTTCATCGATATGTTCGAGGGTATCCAGGACAGCCCCCGCAACTACCAGGAGCTCTGCAACATGTTCAACGATATCTTCCGAAAGGCACCTGTGTACGGTGACCTCGGTCCACCTGTCTACATGATCATGGCCAAGCTGATGAACACCCGAGCAGGTTTCTCCGCATTCACCCGACAGCGACTCAACCTGCATTTCAAGAAGCTCTTCGATACCTGGGGTCTCTTCCTGTCCAGCAAGGATTCCCGCAACGTCCTGGTGGCCGACCAGTTCGACGATCGACACTGCGGTTGGCTCAACGAGCGAGCACTGAGCGCAATGGTGAAGCATTACAACGGTCGCGCCTTCGATGAGGTCTTCCTCTGCGACAAGAACGCCCCATACTACGGCTTCAACTCCTACGACGATTTCTTCAACCGCCGCTTCCGCAACCGAGACATCGATCGACCAGTCGTGGGTGGTGTGAACAACACCACCCTCATCAGCGCAGCATGCGAGTCCCTGAGCTACAACGTCTCCTACGATGTGCAGAGCCTCGACACCCTGGTCTTCAAGGGCGAGACCTACTCCCTGAAGCACCTCCTGAACAACGACCCCTTCACCCCTCAGTTCGAGCATGGCAGCATCCTCCAGGGTTTCCTGAACGTCACCGCATACCACCGATGGCATGCACCTGTCAACGGCACCATCGTGAAGATCATCAACGTCCCAGGTACCTACTTCGCACAGGCACCATCCACCATCGGTGATCCTATCCCAGACAACGATTACGACCCACCCCCTTACCTCAAGTCCCTGGTGTACTTCAGCAACATCGCCGCCCGCCAGATCATGTTCATCGAGGCCGACAACAAGGAGATCGGCCTCATCTTCCTGGTCTTCATCGGTATGACCGAGATCTCCACCTGCGAGGCAACCGTGAGCGAGGGTCAGCACGTCAACCGAGGTGACGATCTCGGCATGTTCCATTTCGGTGGTTCCAGCTTCGCACTCGGTCTGCGAAAGGATTGCCGAGCCGAGATCGTGGAGAAGTTCACCGAGCCAGGTACCGTCATCCGCATCAACGAGGTCGTGGCCGCCCTGAAGGCCTAA |
| *psiK* | ATGGCCTTCGACCTCAAGACCGAGGATGGCCTCATCACCTACCTGACCAAGCACCTCTCCCTGGACGTCGATACCAGCGGTGTGAAGCGCCTGTCCGGCGGTTTCGTCAACGTGACCTGGCGCATCAAGCTCAACGCCCCTTACCAGGGCCATACCAGCATCATCCTGAAGCACGCCCAGCCACATATGTCCACCGACGAGGATTTCAAGATCGGTGTCGAGCGCAGCGTGTACGAGTACCAGGCCATCAAGCTCATGATGGCAAACCGAGAGGTCCTGGGTGGTGTGGACGGTATCGTCTCCGTGCCTGAGGGTCTCAACTACGATCTGGAGAACAACGCCCTCATCATGCAGGACGTCGGCAAGATGAAGACCCTCCTGGATTACGTGACCGCAAAGCCACCTCTCGCAACCGACATCGCACGACTGGTCGGTACCGAGATCGGTGGTTTCGTGGCACGACTGCACAACATCGGTCGAGAGCGCCGCGACGATCCAGAGTTCAAGTTCTTCAGCGGCAACATCGTCGGTCGCACCACCTCCGATCAGCTCTACCAGACCATCATCCCCAACGCCGCCAAGTACGGTGTGGACGATCCACTCCTGCCCACCGTCGTGAAGGACCTGGTCGACGATGTGATGCATAGCGAGGAGACCCTCGTCATGGCCGATCTGTGGTCCGGCAACATCCTCCTGCAGCTCGAGGAGGGTAACCCCAGCAAGCTGCAGAAGATCTACATCCTCGACTGGGAGCTGTGCAAGTACGGCCCTGCCAGCCTCGACCTGGGCTACTTCCTCGGTGATTGCTACCTGATCTCCCGCTTCCAGGATGAGCAGGTCGGTACCACCATGCGACAGGCATACCTCCAGTCCTACGCACGCACCTCCAAGCACAGCATCAACTACGCAAAGGTCACCGCAGGTATCGCAGCACATATCGTGATGTGGACCGACTTCATGCAGTGGGGCAGCGAGGAGGAGCGCATCAACTTCGTCAAGAAGGGTGTGGCAGCATTCCACGACGCACGAGGCAACAACGATAACGGCGAGATCACCTCCACCCTCCTGAAGGAGTCCAGCACCGCCAAGCTTTAA |
| *psiM* | ATGCACATCCGCAACCCATACCGCACCCCCATCGACTACCAGGCACTGTCCGAGGCATTCCCACCTCTCAAGCCATTCGTCAGCGTGAACGCAGACGGTACCTCCAGCGTCGATCTGACCATCCCAGAGGCACAGCGAGCATTCACCGCAGCACTCCTGCATCGAGACTTCGGTCTGACCATGACCATCCCAGAGGATCGACTCTGCCCTACCGTCCCAAACCGCCTGAACTACGTGCTCTGGATCGAGGACATCTTCAACTACACCAACAAGACCCTCGGCCTGTCCGACGATCGCCCCATCAAGGGTGTCGATATCGGCACCGGTGCCAGCGCCATCTACCCTATGCTGGCATGCGCACGATTCAAGGCATGGTCCATGGTCGGCACCGAGGTGGAGCGCAAGTGCATCGACACCGCCCGCCTGAACGTCGTGGCCAACAACCTCCAGGACCGCCTCTCCATCCTGGAGACCAGCATCGATGGTCCTATCCTCGTGCCAATCTTCGAGGCCACCGAGGAGTACGAGTACGAGTTCACCATGTGCAACCCACCATTCTACGACGGTGCAGCAGATATGCAGACCAGCGATGCCGCCAAGGGCTTCGGTTTCGGTGTCGGTGCACCTCACTCCGGTACCGTGATCGAGATGTCCACCGAGGGTGGCGAGAGCGCATTCGTCGCACAGATGGTGCGAGAGAGCCTCAAGCTGCGAACCCGATGCCGATGGTACACCTCCAACCTCGGCAAGCTGAAGAGCCTCAAGGAGATCGTCGGTCTCCTGAAGGAGCTCGAGATCTCCAACTACGCCATCAACGAGTACGTCCAGGGTTCCACCCGACGATACGCAGTGGCCTGGAGCTTCACCGATATCCAGCTGCCAGAGGAGCTCTCCCGACCAAGCAACCCTGAGCTGTCCAGCCTCTTCAAGCTTTAA |
| *PcCPR* | ATGGCAAGCAGCAGCAGCGATGTTTTTGTTCTGGGTCTGGGTGTTGTTCTGGCCGCACTGTATATTTTTCGTGATCAGCTGTTTGCAGCAAGCAAACCGAAAGTTGCGCCGGTTAGCACCACCAAACCGGCAAATGGTAGTGCTAATCCTCGTGATTTTATTGCAAAAATGAAACAGGGCAAAAAGCGTATTGTTATTTTTTATGGTAGCCAGACCGGTACCGCAGAAGAATATGCGATTCGCCTGGCAAAAGAAGCGAAACAGAAATTTGGTCTGGCCAGTCTGGTGTGCGATCCGGAAGAATATGATTTTGAAAAACTGGATCAGCTGCCGGAAGATAGCATTGCATTTTTTGTTGTTGCTACGTATGGTGAAGGTGAACCGACGGATAATGCAGTTCAGCTGCTGCAGAATCTGCAGGATGAAAGCTTTGAATTTAGCTCAGGTGAACGTAAACTGAGCGGTCTGAAATATGTTGTATTTGGCCTGGGTAATAAAACCTATGAACATTATAACCTGATCGGTCGTACCGTTGATGCACAGCTGGCAAAAATGGGTGCCATTCGTATTGGCGAACGTGGTGAAGGTGATGATGATAAAAGTATGGAAGAAGATTATCTGGAATGGAAAGATGGTATGTGGGAAGCATTTGCAACAGCAATGGGTGTGGAAGAAGGTCAGGGTGGTGATAGCGCAGATTTTGTTGTTAGCGAACTGGAAAGCCATCCGCCGGAAAAAGTGTATCAGGGTGAATTTAGCGCACGTGCGCTGACCAAAACCAAAGGTATTCATGATGCAAAAAATCCGTTTGCAGCACCGATTGCAGTTGCACGTGAACTGTTTCAGAGCGTTGTTGATCGTAATTGTGTTCATGTTGAATTTAATATTGAAGGTAGCGGTATTACCTATCAGCATGGTGATCATGTTGGTCTGTGGCCGCTGAATCCGGATGTTGAAGTTGAACGTCTGCTGTGTGTTCTGGGTCTGGCAGAAAAACGTGATGCAGTTATTAGCATTGAAAGCCTGGACCCTGCACTGGCAAAAGTTCCGTTTCCGGTTCCGACCACCTATGGTGCAGTTCTGCGTCATTATATTGATATTAGCGCAGTTGCAGGTCGTCAGATTCTGGGTACCCTGAGCAAATTTGCACCGACCCCGGAAGCAGAAGCATTTCTGCGTAATCTGAATACCAATAAAGAAGAATATCATAATGTTGTTGCAAATGGTTGTCTGAAACTGGGTGAAATTCTGCAGATTGCAACCGGTAATGATATTACCGTTCCGCCGACCACCGCAAATACCACCAAATGGCCGATTCCGTTTGATATTATTGTTAGCGCAATTCCGCGTCTGCAGCCGCGTTATTATAGCATTAGCAGCAGCCCGAAAATTCATCCGAATACCATTCATGCAACCGTTGTTGTTCTGAAATATGAAAATGTTCCGACCGAACCGATTCCGCGTAAATGGGTTTATGGTGTTGGTAGCAATTTTCTGCTGAATCTGAAATATGCAGTTAATAAAGAACCGGTTCCGTATATTACCCAGAATGGTGAACAGCGTGTTGGTGTTCCGGAATATCTGATTGCAGGTCCGCGTGGTAGCTATAAAACCGAAAGCTTTTATAAAGCACCGATTCATGTTCGTCGTAGCACCTTTCGTCTGCCGACCAATCCGAAAAGCCCGGTTATTATGATTGGTCCGGGTACCGGTGTTGCACCGTTTCGTGGTTTTGTTCAGGAACGTGTTGCACTGGCACGTCGTAGCATTGAAAAAAATGGTCCGGATAGCCTGGCAGATTGGGGTCGTATTAGCCTGTTTTATGGTTGTCGTCGTAGCGATGAAGATTTTCTGTATAAAGATGAATGGCCGCAGTATGAAGCAGAACTGAAAGGTAAATTTAAACTGCATTGTGCATTTAGCCGTCAGAATTATAAACCGGATGGTAGCAAAATTTATGTTCAGGATCTGATTTGGGAAGATCGTGAACATATTGCAGATGCAATTCTGAATGGTAAAGGTTATGTTTATATTTGTGGTGAAGCAAAAAGCATGAGCAAACAGGTTGAAGAAGTTCTGGCAAAAATTCTGGGTGAAGCAAAAGGTGGTAGCGGTCCGGTTGAAGGTGTTGCAGAAGTTAAACTGCTGAAAGAACGTAGCCGTCTGATGCTGGATGTTTGGAGCTAA |
| *PcCYB5* | ATGAGCGCAGATAAAATTGTGACGCTGGAAGAACTGCGTGCCCATAAAAGCCGCGATAAACTGTATATTCTGATTCATGGCAAAGTGTATAACGTTACCAAATTTCTGGATGAACATCCGGGTGGTGATGAAGTTCTGCTGGCTGAAGGTGGTATGGATGCAACAGAAGCATTTGAAGATGTTGGTCATAGCGATGAAGCACGTGCACTGCTGCCGGGTATGCTGGTTGGTAGTTTTGAAGAAGGTGCACTGAAAAGCGGTGCAGCAAGTGCGCATGGTGCAAAAGTTGCCACAGCAGTTGAACAGGGTAGCAATCTGATGTATTTTGTTCCTCTGGGTCTGCTGGGTGCATATTTTGCATGGCAGTATTATTCAGGTAGTGCATAA |
| *BaTDC* | ATGAGCGAAAACCTGCAGCTGAGCGCAGAAGAAATGCGTCAGCTGGGTTATCAGGCGGTGGATCTGATTATTGATCACATGAACCATCTGAAAAGCAAACCGGTGAGCGAAACCATTGATTCAGATATCCTGCGTAATAAACTGACAGAAAGCATCCCGGAAAATGGCAGCGATCCGAAAGAACTGCTGCATTTTCTGAATCGTAATGTGTTTAATCAGATCACCCATGTTGATCATCCGCATTTTCTGGCCTTTGTTCCTGGCCCGAATAACTATGTTGGTGTTGTTGCAGATTTTCTGGCTTCTGGTTTTAATGTGTTTCCGACGGCGTGGATCGCAGGCGCAGGTGCAGAACAGATTGAACTGACCACCATTAATTGGCTGAAATCAATGCTGGGTTTTCCGGATAGCGCCGAAGGTCTGTTTGTTAGCGGTGGTAGCATGGCAAATCTGACCGCACTGACAGTTGCACGTCAGGCAAAACTGAATAACGATATTGAAAATGCGGTGGTTTATTTTTCAGATCAGACACATTTTTCCGTTGATCGTGCGCTGAAAGTTCTGGGCTTTAAACATCATCAGATTTGTCGTATTGAAACCGATGAACATCTGCGTATTAGCGTTAGTGCCCTGAAAAAACAGATTAAAGAAGATCGTACCAAAGGTAAAAAACCGTTTTGCGTTATTGCAAACGCCGGTACCACCAATTGTGGTGCAGTTGATTCCCTGAATGAACTGGCAGATCTGTGTAACGATGAAGATGTGTGGCTGCATGCCGATGGTAGCTATGGTGCTCCGGCAATTCTGAGCGAAAAAGGCAGCGCAATGCTGCAGGGTATTCATCGTGCAGATAGCCTGACACTGGATCCGCATAAATGGCTGTTTCAGCCGTATGATGTTGGCTGTGTTCTGATTCGTAACTCTCAGTATCTGAGCAAAACGTTTCGTATGATGCCGGAATATATTAAAGATAGCGAAACTAATGTTGAAGGTGAAATTAATTTTGGCGAATGTGGTATTGAACTGTCTCGCCGTTTTCGTGCCCTGAAAGTTTGGCTGAGCTTTAAAGTTTTTGGTGTTGCAGCATTTCGTCAGGCAATCGATCATGGTATTATGCTGGCTGAACAGGTGGAAGCCTTTCTGGGTAAAGCAAAAGATTGGGAAGTGGTTACGCCTGCACAGCTGGGTATTGTTACGTTTCGTTATATTCCAAGTGAACTGGCAAGCACCGATACTATTAATGAAATTAATAAAAAACTGGTTAAAGAAATCACACATCGTGGTTTTGCAATGCTGAGCACTACAGAACTGAAAGAAAAAGTTGTGATTCGCCTGTGTAGTATTAACCCGCGCACCACCACCGAAGAAATGCTGCAGATTATGATGAAAATCAAAGCACTGGCAGAAGAAGTATCAATTAGCTATCCTTGCGTTGCAGAATAG |
| *CrTDC* | ATGGGTAGCATTGATAGCACGAACGTTGCAATGAGCAACAGCCCGGTTGGTGAATTTAAACCGCTGGAAGCAGAAGAATTTCGTAAACAGGCGCATCGTATGGTTGATTTTATTGCCGATTATTATAAAAACGTTGAAACCTATCCGGTTCTGAGCGAAGTTGAACCGGGTTATCTGCGTAAACGTATTCCGGAAACCGCACCGTATCTGCCGGAACCTCTGGATGATATTATGAAAGATATTCAGAAAGATATCATCCCTGGTATGACCAATTGGATGAGCCCTAATTTTTATGCATTTTTTCCTGCAACCGTTAGTTCAGCCGCCTTTCTGGGTGAAATGCTGAGCACCGCACTGAACAGCGTTGGTTTTACATGGGTTAGCAGCCCGGCAGCCACCGAACTGGAAATGATTGTTATGGATTGGCTGGCACAGATCCTGAAACTGCCTAAAAGCTTTATGTTTAGCGGTACCGGTGGTGGTGTTATTCAGAATACCACCTCAGAAAGTATTCTGTGTACAATTATTGCAGCACGCGAACGTGCTCTGGAAAAACTGGGCCCGGATTCAATTGGTAAACTGGTTTGTTATGGTAGTGATCAGACCCATACCATGTTTCCGAAAACCTGTAAACTGGCAGGCATTTATCCTAATAACATTCGTCTGATTCCGACTACCGTTGAAACCGATTTTGGTATTAGTCCTCAGGTGCTGCGTAAAATGGTCGAAGATGATGTTGCAGCAGGTTATGTTCCACTGTTTCTGTGTGCCACCCTGGGTACCACCTCTACCACCGCAACAGATCCGGTTGATTCCCTGTCTGAAATTGCCAATGAATTTGGTATTTGGATTCATGTTGATGCAGCCTATGCCGGCAGCGCCTGTATTTGCCCTGAATTTCGCCATTATCTGGATGGCATTGAACGTGTTGATAGCCTGAGTCTGAGTCCGCATAAATGGCTGCTGGCATATCTGGATTGTACCTGTCTGTGGGTTAAACAGCCGCATCTGCTGCTGCGTGCACTGACAACCAATCCGGAATATCTGAAAAATAAACAGTCTGATCTGGATAAAGTGGTTGATTTTAAAAACTGGCAGATTGCTACCGGTCGTAAATTTCGTTCCCTGAAACTGTGGCTGATTCTGCGCAGCTATGGTGTTGTTAACCTGCAGTCACATATTCGTAGCGATGTTGCGATGGGTAAAATGTTTGAAGAATGGGTTCGTAGCGATAGCCGTTTTGAAATTGTTGTTCCGCGCAATTTTTCTCTGGTTTGTTTTCGTCTGAAACCGGATGTTAGCAGCCTGCATGTTGAAGAAGTTAATAAAAAACTGCTGGATATGCTGAATTCAACCGGTCGTGTTTATATGACCCATACAATTGTTGGTGGTATTTATATGCTGCGCCTGGCAGTTGGTTCAAGCCTGACCGAAGAACATCATGTTCGCCGTGTTTGGGATCTGATCCAGAAACTGACCGATGATCTGCTGAAAGAAGCGTAG |
| *PacPsiM* | ATGCACAACCGCAATCCGTACCGTGATGTTATCGATTACCAGGCACTGGCGGAAGCCTATCCGCCACTGAAACCGCACGTAACCGTTAACGCGGACAACACCGCAAGCATCGACCTGACCATTCCGGAAGTGCAGCGCCAGTATACCGCGGCATTGCTGCACCGCGACTTCGGTCTGACCATCACTCTGCCGGAAGATCGCCTGTGTCCGACCGTACCAAACCGTCTGAACTACGTACTGTGGATCGAAGACATCTTCCAGTGCACTAACAAGGCTCTGGGTCTGTCCGACGACCGTCCGGTTAAAGGTGTAGACATCGGTACCGGCGCATCTGCAATCTACCCGATGCTGGCTTGCGCGCGTTTCAAACAGTGGTCCATGATCGCTACCGAAGTAGAGCGTAAATGCATCGACACCGCGCGTCTGAACGTGCTGGCTAACAACCTGCAAGACCGTCTGTCCATTCTGGAAGTATCCGTAGACGGTCCGATTCTGGTACCGATCTTCGACACTTTCGAACGTGCGACCTCTGATTATGAGTTCGAGTTCACCATGTGTAACCCGCCGTTCTACGATGGTGCTGCTGACATGCAGACCTCCGATGCGGCCAAAGGTTTCGGTTTCGGTGTTAACGCTCCGCACTCTGGCACCGTGATCGAAATGGCAACCGAAGGTGGTGAAGCGGCGTTCGTTGCACAGATGGTACGTGAATCTATGAAACTGCAGACTCGTTGCCGTTGGTTCACCTCCAACCTGGGCAAACTGAAGAGCCTGCACGAAATCGTAGCTCTGCTGCGTGAAAGCCAGATCACCAACTACGCTATCAACGAATATGTGCAGGGTACGACTCGTCGTTATGCTCTGGCTTGGAGCTTTACCGACATCAAACTGACCGAAGAACTGTACCGTCCGAGCAATCCGGAACTGGGTCCGCTGTGCTCCACCTTCGTGTAA |
| *GdPsiM* | ATGCACATCCGTAATCCGTACCTGACTCCGCCAGACTACGAAGCTCTGGCTGAAGCGTTCCCGGCGCTGAAACCGTACGTTACCGTTAATCCAGACAAGACCACCACCATCGACTTCGCGATTCCGGAAGCTCAGCGTCTGTACACCGCTGCGCTTCTGTACCGTGACTTCGGTCTGACCATCACCTTGCCGCCGGACCGTCTGTGTCCGACTGTTCCAAACCGTCTGAACTACGTTCTGTGGATTCAGGACATTCTGCAGATCACCTCTGCAGCGCTGGGTCTGCCAGAAGCTCGTCAGGTTAAAGGTGTTGACATCGGTACTGGTGCGGCGGCTATCTATCCGATCTTGGGTTGCTCTCTGGCGAAGAACTGGTCTATGGTTGGTACTGAAGTTGAACAGAAATGCATCGACATCGCTCGTCAGAACGTTATCTCTAACGGTCTGCAAGACCGTATCACCATCACCGCGAACACCATCGACGCTCCGATCTTGCTGCCGCTGTTCGAAGGTGACTCTAACTTCGAATGGGAGTTCACCATGTGCAATCCACCGTTCTACGATGGTGCGGCTGACATGGAAACCTCTCAAGACGCGAAAGGTTTCGGTTTCGGTGTTAACGCGCCGCACACCGGTACCGTTGTTGAAATGGCGACTGATGGTGGTGAAGCAGCGTTCGTTTCTCAGATGGTTCGTGAATCTCTGCACCTGAAGACTCGTTGCCGTTGGTTCACCTCTAACCTGGGTAAACTGAAGAGCCTGCACGAAATCGTTGGTCTGCTGCGTGAACACCAGATCACCAACTACGCGATCAACGAATACGTTCAGGGTACCACTCGTCGTTACGCTATCGCGTGGTCTTTCACCGATCTGCGTCTGTCTGACCACCTGCCGCGTCCACCAAATCCGGACCTGTCTGCGCTGTTCTAA |
| *PscPsiM* | ATGCACATCCGCAATCCGTACCGTTCTCCGATCGACTACCAGGCTCTGGTTGAAGCATTCCCGCCGCTGCGTCCGTACGTTACCGTAAACCAAGACAACACCACTTCCATCGACCTGACTGTTCCGGAAGTTCAGCGTCTGTACACCGCTGCTCTGCTGCATCGTGACTTCGGTCTGGTTATCGACCTGCCAGAAGATCGTCTGTGTCCGACTCTGCTGACTCGTACGCCGCGTCTGAACTATGTGTTGTGGGTGGAAGACATCTTGAAAGTAACCAACACCGCGCTGGGTCTGAGCGAAGACCGTCCGGTTAAAGGCATCGATATCGGTACCGGTGCTGCGGCGATCTATCCGATGCTGGCTTGCGCTCGTTTCAAGACCTGGTCTATGATCGGTACTGAAATCGACCGTAAATGTATCGACACCGCACGTGTTAACGTTCTGACTAACAACCTGCAAGACCGTCTGTCCATCATCGAAACCTCTATCGACGGTCCGATCTTGGTACCGATCTTCGAAGCAACCACCGACTACGAATACGACTTCACCATGTGTAACCCGCCATTCTACGATGGTGCTGCTGACATGCAGACTTCCGACGCTGCGAAAGGTTTCGGTTTCGGTGTTAACGCACCACACTCTGGTACTGTTATCGAAATGTCTACCGAAGGTGGTGAAAGCGCGTTCGTTGCTCAGATGGTTCGTGAATCTCTGGACCACCGTACTCGTTGCCGTTGGTTCACCTCCAACCTGGGTAAACTGAAATCTCTGCACGAAATCGTTGGTTTGCTGCGTGAACACCAGATCTCTAACTACGCAATCAACGAATATGTTCAGGGTACCACGCGTCGTTACGCGATCGCTTGGAGCTTCACCAACATCCGTCTGCCGGAAGACCTGACTCGTCCGTCCAATCCGGAACTGTCTAGCTTGTTCTAA |

Supplementary Figures


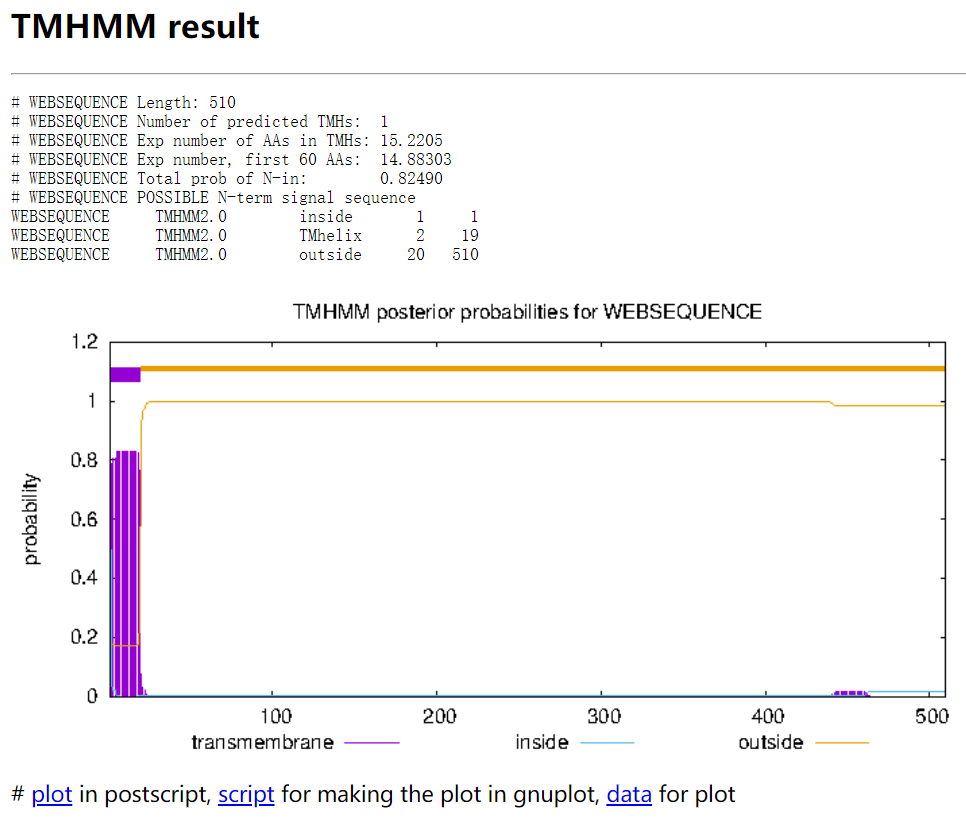


**Figure S1** **Prediction of the transmembrane region of PsiH using TMHMM v2.0.**





**Figure S2 The catalytic ability of PsiH variants.** (A) HPLC analysis of the extracts from the *E. coli* BL21 strains expressing different PsiH variants supplemented with tryptamine. (B) HRMS analysis of the product 4-hydroxytryptamine. (C) The titers of 4-hydroxytryptamine produced by trPsiH at different temperatures. Error bar represents the standard deviation of three independent biological replicates. Significance analysis was performed by one-way ANOVA. Significant difference is labelled with asterisk (*** *p* < 0.001, ** *p* < 0.01, * *p* < 0.05), and no significant difference is indicated by “ns”.





**Figure S3 The catalytic ability of different tryptophan decarboxylases.** (A) HPLC analysis of the extracts from the *E. coli* BL21 strains expressing different tryptophan decarboxylases supplemented with tryptophan. (B) The titers of tryptamine produced by different tryptophan decarboxylases at different temperatures. Error bar represents the standard deviation of three independent biological replicates. Significance analysis was performed by one-way ANOVA. Significant difference is labelled with asterisk (*** *p* < 0.001, ** *p* < 0.01, * *p* < 0.05), and no significant difference is indicated by “ns”.





**Figure S4 The catalytic ability of PsiK and PsiM.** (A) HPLC analysis of the extracts from the *E. coli* BL21 strain expressing *psiK* and *psiM* genes supplemented with 4-hydroxytryptamine. (B) The titers of psilocybin produced by the strain expressing *psiK* and *psiM* genes at different temperatures. Error bar represents the standard deviation of three independent biological replicates.





**Figure S5** **LC-HRMS analysis of products produced by strain P03.** (A) The extracted ion chromatogram of [M+H]^+^ = 257, 271, 285, 191 and 205 indicates norbaeocystin, baeocystin, psilocybin, norpsilocin and psilocin, respectively. (B) MS spectra of norpsilocin ([M+H]^+^ *m/z*: calculated 191.1184, observed 191.1184). (C) MS spectra of psilocin ([M+H]^+^ *m/z*: calculated 205.1341, observed 205.1340).





**Figure S6 PCR validation of the gene knockout *E. coli* strains.** (A) Schematic illustration of *tnaA* gene knockout using the CRISPR/Cas9-mediated gene editing strategy. (B) Schematic illustration of *trpR* gene knockout using the CRISPR/Cas9-mediated gene editing strategy. (C) PCR validation of the strain △tnaA. The *tnaA* gene in strains from line 1-7 was all successfully knocked out. (D) PCR validation of the strain △trpR. The *trpR* gene in strain from line 5 was successfully knocked out.


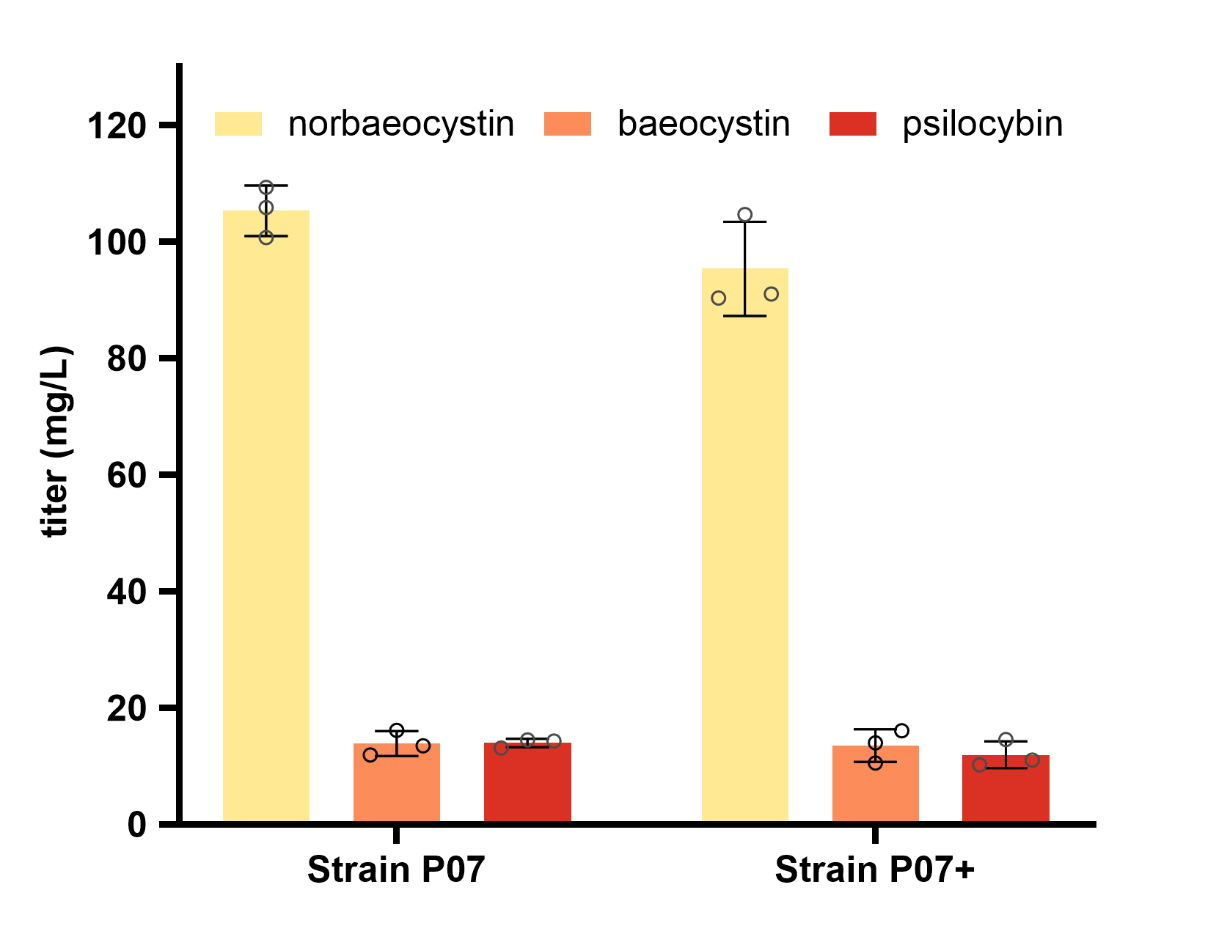
**Figure S7 The titers of psilocybin produced in the strain P07 with or without feeding methionine.** Strain P07: without feeding methionine; Strain P07+: feeding with 0.5 mg/mL methionine Error bar represents the standard deviation of three independent biological replicates.





**Figure S8 Mining of PsiM homologues.** The phylogenetic analysis of PsiM homologues obtained from the NCBI database. Three PsiM homologues (Table S4) located in the blue colored branch were selected for the catalytic assay. They were PacPsiM from *Panaelus cyanescens* (PPQ80976.1), GdPsiM from *Gymnopilus dilepis* (PPQ70884.1), and PscPsiM from *Psilocybe cyanescens* (PPQ83230.1), respectively.





**Figure S9 The titers of psilocybin produced in the strains expressing different PsiM homologues.** Strain P07: PsiM from *P. cubensis*; Strain P10: PacPsiM from *P. cyanescens*; Strain P11: GdPsiM from *G. dilepies*; Strain P12: PscPsiM from *P. cyanescens*; Error bar represents the standard deviation of three independent biological replicates.





**Figure S10 Summary of the** **standard curves.** (A) The standard curve of tryptamine. (B) The standard curve of psilocybin. Tryptamine and 4-hydroxytryptamine were quantified using the standard curve of 5-hydroxytryptamine. Baeocystin, norbaeocystin and psilocybin were quantified using the standard curve of psilocybin.

**Reference**

Jiang Y, Chen B, Duan C, Sun B, Yang J, Yang S (2015) Multigene editing in the

*Escherichia coli* genome via the CRISPR-Cas9 system. *Appl. Environ. Microbiol.,*

81(7):2506-2514.
